# Cholera diagnosis in human stool and detection in water: A systematic review and meta-analysis

**DOI:** 10.1371/journal.pone.0270860

**Published:** 2022-07-06

**Authors:** Jennifer Falconer, Karin Diaconu, Fiona O’May, Advaith Gummaraju, Ifeyinwa Victor-Uadiale, Joseph Matragrano, Berthe-Marie Njanpop-Lafourcade, Alastair Ager

**Affiliations:** 1 Institute for Global Health and Development, Queen Margaret University, Edinburgh, Scotland; 2 London School of Hygiene and Tropical Medicine, London, United Kingdom; 3 Columbia University, New York, New York, United States of America; 4 Agence de Médecine Préventive, Paris, France; 5 Mailman School of Public Health, Columbia University, New York, New York, United States of America; Stellenbosch University, SOUTH AFRICA

## Abstract

**Background:**

Cholera continues to pose a problem for low-resource, fragile and humanitarian contexts. Evidence suggests that 2.86 million cholera cases and 95,000 deaths due to cholera are reported annually. Without quick and effective diagnosis and treatment, case-fatality may be 50%. In line with the priorities of the Global Task Force on Cholera Control, we undertook a systematic review and meta-analysis of diagnostic test accuracy and other test characteristics of current tests for cholera detection in stool and water.

**Methods:**

We searched 11 bibliographic and grey literature databases. Data was extracted on test sensitivity, specificity and other product information. Meta-analyses of sensitivity and specificity were conducted for tests reported in three or more studies. Where fewer studies reported a test, estimates were summarised through narrative synthesis. Risk of Bias was assessed using QUADAS-2.

**Results:**

Searches identified 6,637 records; 41 studies reporting on 28 tests were included. Twenty-two tests had both sensitivities and specificities reported above 95% by at least one study, but there was, overall, wide variation in reported diagnostic accuracy across studies. For the three tests where meta-analyses were possible the highest sensitivity meta-estimate was found in the Cholera Screen test (98.6%, CI: 94.7%-99.7%) and the highest specificity meta-estimate in the Crystal VC on enriched samples (98.3%, CI: 92.8%-99.6%). There was a general lack of evidence regarding field use of tests, but where presented this indicated trends for lower diagnostic accuracy in field settings, with lesser-trained staff, and without the additional process of sample enrichment. Where reported, mean test turnaround times ranged from over 50% to 130% longer than manufacturer’s specification. Most studies had a low to unclear risk of bias.

**Conclusions:**

Currently available Rapid Diagnostic Tests can potentially provide high diagnostic and detection capability for cholera. However, stronger evidence is required regarding the conditions required to secure these levels of accuracy in field use, particularly in low-resource settings.

**Registration:**

PROSPERO (CRD42016048428).

## Introduction

Cholera is caused by the etiological agents *Vibrio cholerae* O1 and O139 serogroups and transmitted through the faecal-oral route by consuming contaminated food or water [1, 2]. The predominant symptom is acute watery diarrhoea, which may be like rice-water, and sometimes accompanied by vomiting; although only 20% of those infected develop these symptoms. Without treatment with oral and intravenous rehydration therapy, case fatality may be as high as 50% [3].

While recent estimates are not available, earlier evidence suggests that in endemic countries, cholera is responsible for an estimated 2.86 million cases, and 95,000 deaths annually, with 1.3 billion people at risk [4]. Figures are likely to be an underestimate given inadequate surveillance systems, diagnostic tests, and a reluctance by authorities to acknowledge outbreaks [5]. The heaviest burden occurs in sub-Saharan Africa and South Asia, but the disease is endemic in Africa, Asia, South America, and Central America [4, 6]. Epidemics occur frequently in fragile and conflict-affected states, where the logistical coordination of intervention delivery is complex. In the past ten years large outbreaks have been seen in countries including Yemen, Haiti, Iraq, Sierra Leone, Somalia, South Sudan, Tanzania, and Zimbabwe [7, 8]. The Yemen outbreak between April 2017 and October 2018 was the largest ever epidemiologically seen, with over 1.2 million cases reported, of which 2,556 cases proved fatal [9].

Diagnosis and detection in the early stages of an epidemic are essential for outbreak confirmation and control, and identification of areas for targeted interventions to control disease spread [5, 10]. Bacterial culture continues to be considered the gold standard for diagnosis of cholera–from both water or stool samples, yet suffers from issues surrounding precision, sample transport, laboratory infrastructure, a time delay of two to three days, and necessity of highly trained laboratory technicians [1, 10]. Use of polymerase chain reaction (PCR) to detect cholera is becoming increasingly common, yet, like culture, requires a laboratory and highly trained staff, both of which are sparsely available in the settings in which cholera outbreaks are most common [10, 11].

The Global Task Force on Cholera Control highlights early identification as critical to cholera detection [12] and Rapid Diagnostic Tests (RDTs) are widely viewed as a pragmatic alternative to laboratory-based detection methods [5]. For humanitarian response, detection in both water samples and stool is particularly promising [12].

RDTs potentially provide a cheap, accurate, quick, easy to use, and robust diagnostic tool [10, 11]. Over the past three decades a number of such tests have been developed and validated–e.g., Crystal VC, and the Institut Pasteur (IP) dipstick–in field settings including Bangladesh, Guatemala, Mexico, and Mozambique [11]. A previous review on the topic carried out in 2012 by Dick et al. [11] identified 24 cholera diagnostic tests, including RDTs, PCR technologies, agglutination, and direct fluorescence antibodies. Turnaround time of these tests was as little as 15 minutes. However, diagnostic accuracy of these RDTs for individual patients was variable; reported sensitivities ranged from 58–100%, and specificities from 60–100%. Additionally, the quality of the 18 peer-reviewed articles included in the review was found to be low, with issues surrounding sample size and sample types, the context of field-tests, and gold standards [11]. More recently, two reviews of methods for detecting cholera have been published [13, 14]. These both cover laboratory tests and field-based RDTs and focus on the technical mechanisms by which these different tests work. Ramamurthy and colleagues [13] particularly highlight new methods such as loop-mediated isothermal amplification (LAMP) with the use of a lateral chromatographic flow dipstick and use of genome sequencing data, which show promising results for detection of cholera–however research on these in field settings is currently limited.

While these three reviews provide a scope of the field of cholera diagnostic tests, they are not systematic reviews, and did not review the literature using rigorous search methods, nor do they undertake any meta-analysis. Finally, critical information on product design, pricing, ease of use and training requirements were missing from these reviews–this information is highly pertinent given the low-resource settings in which these products are most needed.

Evidence surrounding accurate cholera diagnosis, and in particular rapid diagnostic tests, remains highly topical, with recent reviews suggesting that such tests still see limited use for either surveillance or outcome detection [15]. This study aimed to appraise the evidence of diagnostic accuracy and other features relevant to use in low-income settings (such as pricing and design features) of current cholera diagnosis and detection tests for use with water or stool samples. This analysis is clearly relevant in the assessment of the suitability of current diagnostic tests for the wider use in such settings required to meet roadmap goals. Further, noting the emergence of novel diagnostic technologies [16, 17], it is also relevant in informing the target product profile required of any new product proposed for use at scale in low-resource contexts in this field.

## Methods

A systematic review and meta-analysis were undertaken to identify current products for cholera diagnosis or detection in stool or water samples. The review was completed according to the Preferred Reporting Items for Systematic Reviews and Meta-analysis of Diagnostic Test Accuracy (PRISMA-DTA) guidelines [18]. A completed PRISMA-DTA checklist is available in S1 Appendix.

A protocol for this study was published in 2018 [16] and the review is registered with PROSPERO (CRD42016048428).

### Search strategy and study selection

Eleven databases, comprising both peer-reviewed and grey literature, were searched: MEDLINE, Embase, CINAHL, Scopus, ProQuest, Global Health Library (WHOLIS), IndMed, OpenGrey, WHO IRIS, ClinicalTrials.gov, and ICTRP WHO. A full list of search strategies can be found in S2 Appendix. Initial searches were carried out in October 2017 and updated in March 2020. Reference lists of included studies were scanned for additional relevant records.

Searches were undertaken by one reviewer (JF) and screened by two reviewers (JF and KD). Discrepancies were resolved through discussion, and by an arbiter (JM) where no consensus could be reached.

### Inclusion and exclusion criteria

Study inclusion was determined according to the following criteria:

#### Population

People suspected to be infected with cholera.

#### Index test

Diagnostic tests developed for rapid use with field samples.

#### Target condition

Detection of *V*. *cholerae* in human stool and water.

#### Reference test

Culture or PCR, or a combination reference including one of these.

#### Setting

Field or laboratory setting.

#### Outcome

Sensitivity and specificity.

We included primary field and laboratory evaluations of any study design that compared a test for cholera to a reference test, validated using field samples of water or human stool. We excluded studies which used only artificially created cholera samples and studies without a non-cholera control–i.e., that only included samples positive for cholera. We also excluded abstracts and articles with insufficient information on our review objective, non-research reports, opinions, editorials, and modelling studies.

No restrictions were placed upon publication date, language, or location of study.

### Data extraction and analysis

In line with the protocol, analysis of studies included descriptions of:

- Diagnostic accuracy of the products–e.g., sensitivity and specificity
- Technical characteristics of products–e.g., detection target and turnaround time
- Information on product pricing and ease of use

Positive and negative predictive values (PPV and NPV) were intended for inclusion as per the study protocol, however, due to inconsistencies in reporting PPV and NPV, we focussed solely on sensitivity and specificity.

Information was therefore extracted from papers on: study characteristics; product specifications of diagnosis and detection technologies; sample characteristics, preparation, and handling; outcome measures including sensitivity, specificity, true positives, false positives, true negatives, false negatives; and data on test pricing, design characteristics, and ease of use. Data extraction was performed in duplicate using an extraction sheet designed and piloted prior to study selection. One reviewer (JF) extracted all papers, with second extraction done by a team of three reviewers (KD, FO’M and AG). Discrepancies were resolved by an independent arbiter (IV).

Study quality and bias were assessed using the QUADAS-2 tool for studies of diagnostic accuracy [19]. Risk of bias was assessed by two reviewers (JF and KD). QUADAS-2 questions were focused on assessing both risk of bias and applicability and in the case of laboratory studies were adapted to include consideration of both patient and sample selection. Where no information was presented at all or insufficient information was available to reach judgment, we noted answers to QUADAS-2 as unclear. Where information was available, and studies used good practice in line with other studies of diagnostic accuracy, and concerns over test applicability were not present, we judged risk of bias and concerns over applicability as low. To reach judgments on applicability, we considered all relevant test characteristics discussed in the document review–i.e., both issues of diagnostic accuracy, but also intended use of test as described by authors, including relevant information on technical specifications and cost of test among others. No formal assessment of publication bias was conducted.

#### Meta-analysis

Meta-analyses of sensitivity and specificity were undertaken according to the methods outlined in Shim et al., 2019 [20]. Meta-analyses were carried out where data was available for three or more studies testing on the same sample type (i.e., stool or water), with the same sample handling (i.e., direct versus enriched samples). Raw numbers of true positives, false positives, true negatives, and false negatives were required, so studies without this information were excluded from meta-analysis. A separate meta-analysis was carried out for each reference test where these criteria were met.

A random effects model was used to account for variation across studies, and forest plots were produced to provide a visual depiction of variability. To avoid sample overlap, only one estimate of sensitivity and one estimate of specificity was included per study in each meta-analysis. The one exception to this was where studies reported separate estimates by geographical location. Where studies had more than one estimate calculated based on the same samples (e.g., due to lab technicians and field technicians both undertaking the test), priority was given to results obtained from settings most similar to that intended by the test.

Due to the correlation of sensitivity and specificity estimates, additional analyses were undertaken to overcome this and provide single meta-estimates of diagnostic accuracy.

Summary receiver operating characteristic (SROC) curves of sensitivity against false positive rate (false positive rate = 1-specificity) were plotted and the area under the curve (AUC) calculated. The AUC varies from 0 to 1 and estimates the percentage of correct predictions of a test, with a value of 0 representing a test whose diagnoses are 100% wrong, 1 representing a test whose diagnoses are 100% correct, and 0.5 representing a test with a 50% chance of a correct diagnosis.

Additional supplementary meta-analyses were undertaken using diagnostic odds ratios (DOR). Further details can be found in S3 Appendix.

#### Narrative synthesis

Given meta-analysis was not possible for the majority of tests, sensitivity and specificity results were also synthesized narratively, by presenting a range of estimates for each test, and plotting sensitivities and specificities graphically. Tests were sorted into three groups for narrative synthesis: immunologically-based tests, PCR-based tests, and ‘other’ test types. Results of studies were sub-grouped by intended location of test, sample type, target, and whether the sample was enriched prior to testing. For each of these groups the range of sensitivities and specificities reported in studies is detailed. Tests were classified as laboratory evaluations or field evaluations according to a) the location where the test was undertaken and b) the personnel who undertook the test (for example field technicians or clinicians, versus lab technicians). This classification was undertaken by reviewers based on the descriptions of test procedures available in the included texts, and the assessment of this review may deviate from the ‘field’ or ‘laboratory’ label used by the authors of a study.

Information on other components of the diagnostic products was also synthesized narratively, within the same three groups. This means that where available we extracted and report on the information study authors provided regarding the way in which products are due to be used (including ease of use, necessary training of health care workers, instructions for use) and their potential value for money (e.g., elements of cost, efficiency of deployment).

Meta-analysis was undertaken in R version 3.6.3, and all other analysis was undertaken in Microsoft Excel.

## Results

### Search results

**Fig 1:**
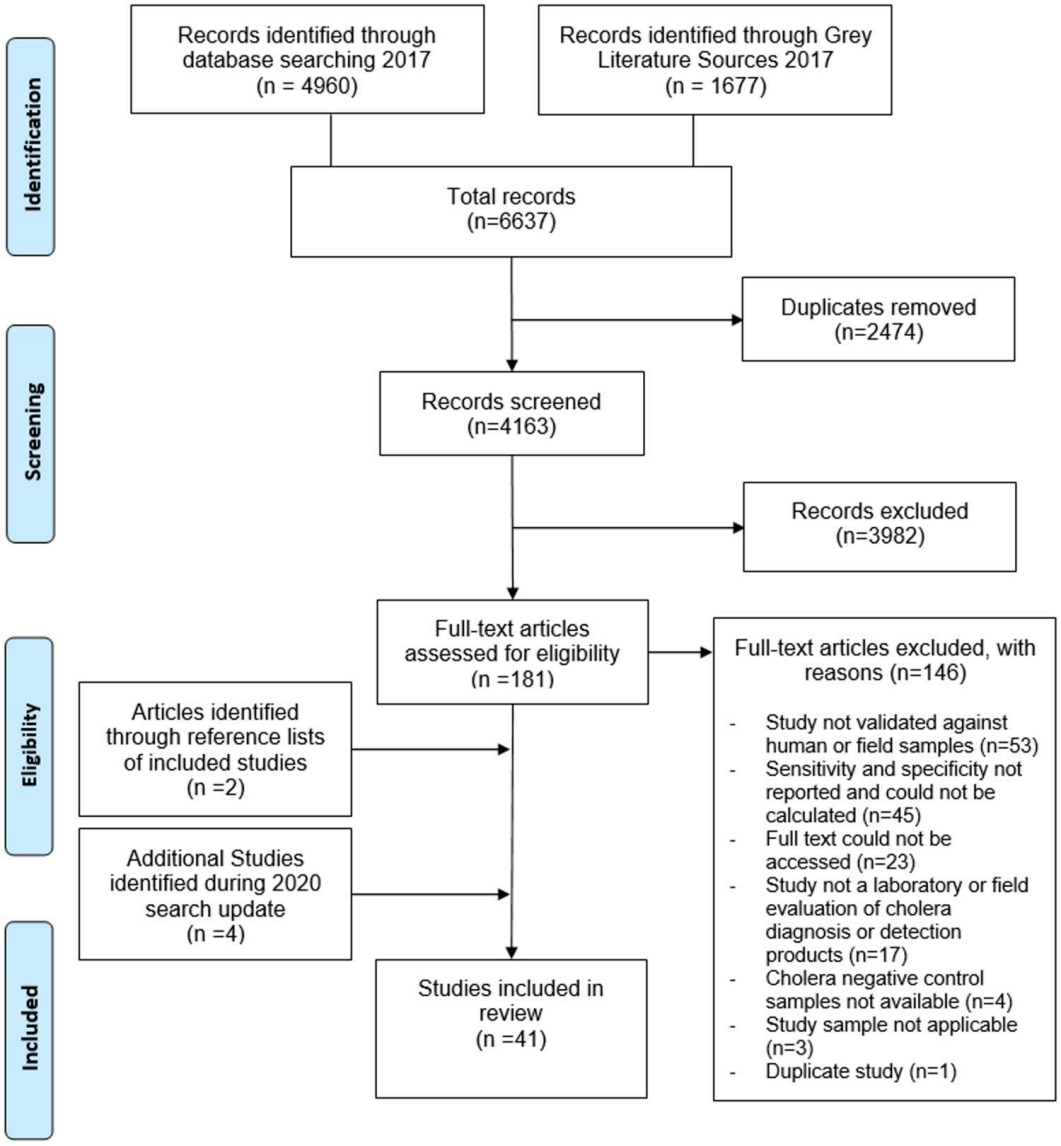
PRISMA flow chart.

Searches identified 6,637 records. Once duplicates were removed, the titles and abstracts of 4,163 records were screened for relevance. Full text review was undertaken on 181 papers, and 35 were selected for analysis. The search process is detailed in Fig 1, including the reasons for exclusion of 146 records during the full text assessment.

During search updates in March 2020, a further 602 records were retrieved (after exclusion of duplicates with original search), and four additional studies identified for inclusion. Two further studies were identified through reference lists of included studies, resulting in 41 studies in total being included in the final analysis.

### Characteristics of included studies

The 41 studies included 13 field assessments and 31 laboratory assessments of cholera diagnostic products (four studies included both field and laboratory assessments; one study was unclear on the location). Samples came from a range of countries, primarily in South and East Asia, the most reported being Bangladesh (13 studies) and India (eight studies). Twenty-eight different products were reported on. These included immunologically-based tests detecting lipopolysaccharides or proteins of *V*. *cholerae* (for example Crystal VC, Cholera SMART, and Cholera Screen), and PCR-based tests detecting genes or nucleic acids (for example TaqMan Array Card). Most studies utilised stool samples in their testing, however five studies using water samples were included. Reference tests were overwhelmingly bacterial culture (in 33 studies), with PCR (in six studies), or combination references (three studies) also used. Overall, 24,835 samples were captured, with individual study sample sizes ranging from 27 to 6,497. Complete study characteristics are presented in Table 1.

**Table 1:** Characteristics of included studies.

Study ID	Country	Index test(s)	Intended test location	Location assessed by study	Reference standard(s)	Sample type	Sample size
Albert 1997 [21]	Not specified	PCR assay (with new primers O139-1 and O139-2)	Laboratory	Laboratory	Culture	Stool	180
Bhuiyan 2003 [22]	Bangladesh	IP dipstick	Field	Laboratory	Culture	Stool	134
Bolaños 2004 [23]	Costa Rica	Cholera SMART	Field	Laboratory	Culture	Stool	28^2^
		Pathogen Detection Kit (PDK)	Field	Laboratory	Culture	Stool	27^2^
Bwire 2017 [24]	Uganda	Crystal VC	Field	Laboratory	Culture	Stool	102
Carillo 1994 [25]	Peru	Cholera Screen	Field	Laboratory	Culture	Stool	100
		Latex agglutination test	Field	Laboratory	Culture	Stool	99
Chaicumpa 1995 [26]	Thailand	Dot-blot ELISA	Laboratory	Laboratory	Culture	Stool	984
Chaicumpa 1998 [27]	Thailand and India	Dot-blot ELISA	Laboratory	Laboratory	Culture	Stool	6497
Chakraborty 2013 [28]	Bangladesh	Crystal VC	Field	Laboratory	Culture	Water	550
Colwell 1992 [29]	Guatemala	Cholera Screen	Field	Laboratory and Field	Culture	Stool	17
	Bangladesh	Cholera Screen	Field	Laboratory and Field	Culture	Stool	77
Debes 2016 [30]	Cameroon	Crystal VC	Field	Field	PCR	Stool^1^	673
Eddabra 2011 [31]	Laboratory in France; samples from Ivory Coast	ChromID Vibrio	Laboratory	Laboratory	Combination (by Vitek 2 and/or ID 32 E biochemical strips, PCR, or comparison of ChromID Vibrio with TCBS)	Stool	30
		TCBS	Laboratory	Laboratory	As above	Stool	30
George 2014 [32]	Bangladesh	Crystal VC	Field	Field and laboratory	Culture	Stool	125
Hao 2017 [33]	China	Vch-UPT-LF	Field	Laboratory	Combination (culture or 2+ of colloidal gold assay, real time fluorescent PCR, Vch-UPT-LF)	Water	102
Harris 2009 [34]	Guinea-Bissau	Crystal VC	Field	Field	PCR	Stool	101
Hasan 1994a [35]	Bangladesh	Cholera DFA	Laboratory	Laboratory	Culture	Stool	44
Hasan 1994b [36]	Bangladesh	Cholera SMART	Field	Field	Culture	Stool	44
	Mexico	Cholera SMART	Field	Field	Cholera Screen	Stool	108
Hasan 1995 [37]	India^3^	BengalScreen	Laboratory	Laboratory	Culture	Stool^1^	35
	India^3^	Bengal DFA	Laboratory	Laboratory	Culture	Stool^1^	35
Hoshino 1998 [38]	India	Multiplex PCR (with: O139-rfb primers—O139F-2, O139R-2; O1-rfb primers—O1F-2, O1R-2; cholera toxin primers—VCT1, VCT2)	Laboratory	Laboratory	Culture	Stool	121
Islam 1994 [39]	Bangladesh	Cholera Screen	Field	Laboratory	Culture	Stool	57
Islam 2019 [40]	Bangladesh	Crystal VC	Field	Field	Culture	Stool	5865
		Cholkit	Field	Field	Culture	Stool	1355
Jin 2013 [41]	China	CT-RTCA (cholera toxin real-time cell analysis)	Laboratory	Laboratory	Combination (two or more matching results among real-time PCR, VET-RPLA, and CT-RTCA results)	Stool	100
Kalluri 2006 [42]	Bangladesh	Cholera SMART	Field	Field and laboratory	Culture	Stool	304
		IP dipstick	Field	Field and laboratory	Culture	Stool	304
		Medicos dipstick	Field	Field and laboratory	Culture	Stool	304
Ley 2012 [43]	Zanzibar	Crystal VC	Field	Field and laboratory	Culture	Stool	622
Liu 2013 [44]	Laboratory in USA; clinical samples from Tanzania and Bangladesh	TaqMan Array Card (singleplex real time PCR format)	Laboratory	Laboratory	Conventional assay and PCR-Luminex.	Stool	189
Matias 2017 [45]	Haiti	Crystal VC	Field	Laboratory	Culture	Stool	511
		Artron RDT	Field	Laboratory	Culture	Stool	129
		SD Bioline	Field	Laboratory	Culture	Stool	451
Momtaz 2013 [46]	Iran	PCR (with primers specific to espM gene)	Laboratory	Laboratory	Culture	Water	448
Mukherjee 2010 [47]	India	Crystal VC	Field	Field	Culture	Stool	212
Mwaba 2018 [48]	Zambia	SD Bioline	Field	Field	Culture and PCR	Stool	170
Nato 2003 [49]	Madagascar	IP dipstick	Field	Laboratory	Culture	Stool	140
	Bangladesh	IP dipstick	Field	Laboratory	Culture	Stool	102
Ontweka 2016 [10]	South Sudan	Crystal VC	Field	Field	PCR	Stool	101
Page 2012 [50]	Democratic Republic of Congo	Crystal VC	Field	Field	Culture or Culture and PCR	Stool	256
Qadri 1994 [51]	Bangladesh	Co-agglutination test (COAT)	Field	Laboratory	Culture	Stool	230
Qadri 1995 [52]	Bangladesh	Bengal SMART	Field	Laboratory	Culture	Stool	189
Ramamurthy 1992 [53]	India	Bead ELISA	Laboratory	Laboratory	Culture	Stool	75
Ramamurthy 1993 [54]	India	Bead ELISA and PCR (with primers specific to V. cholerae O1)	Laboratory	Laboratory	Culture	Stool	123
Ramamurthy 1996 [55]	India	Bead ELISA	Laboratory	Laboratory	Culture	Stool	95
Rashid 2017 [1]	Bangladesh	Crystal VC	Unclear	Laboratory	Culture	Water	1648
Sayeed 2018 [56]	Bangladesh	Cholkit	Field	Laboratory	Culture	Stool	76
		Crystal VC	Field	Laboratory	Culture	Stool	76
		Multiplex PCR (with: O139-rfb primers—O139F-2, O139R-2; O1-rfb primers—O1F-2, O1R-2; cholera toxin primers—VCT1, VCT2)	Laboratory	Laboratory	Culture	Stool	76
Supawat 1994 [57]	Thailand	Cholera diagnostic kit (mAb-based dot-blot ELISA)	Field	Field	Culture	Stool	211
Tuteja 2007 [58]	India	Two-tip dipstick sandwich ELISA	Unclear	Unclear	Culture.	Water	50
						Stool	75
Wang 2006 [59]	Mozambique	IP dipstick	Field	Laboratory	Culture	Stool	391

^1^Water also tested, but sensitivity and specificity data not available

^2^For purposes of this review, ’high probability samples’ were included in analysis

^3^Results from Mexico excluded, due to absence of any cholera negative samples

The 28 different diagnostic tests and detection products reported included several different types of test. For the purposes of analysis, these were split into three broad categories, based on the mechanism of action of the diagnostic test: ‘immunologically-based’ tests detecting lipopolysaccharides or proteins, PCR-based tests, and ‘other tests’–which included selective media-based tests and real-time cell analysis. Immunologically-based tests are those detecting antigens of *V*. *cholerae* O1 and O139, such as lipopolysaccharides or proteins (reported in studies in a variety of ways, for instance ‘*V*. *cholerae* O1 antigen A’ [57], ‘‘A’ factor of *V*. *cholerae* O139’ [52], ‘V. *cholerae* O1 and O139 antigens’ [34]). These tests were predominantly intended for field use (17 of 20 tests). PCR-based tests are those detecting genes and nucleic acids associated with pathogenic *V*. *cholerae* through PCR, for instance detecting the toxR gene [44], or espM gene [46] of *V*. *cholerae*. Finally, other types of tests included selective media-based tests, and real-time cell analysis. In selective media-based tests, stool samples containing bacterial organisms–in this case *V*. *cholerae*–were grown on selective medium and preliminary identified on the basis of colony appearance [31]. The single cholera-toxin real-time cell analysis test included assesses how different mammalian cell types respond to cholera toxin as they grow [41].

### Results: Meta-analysis

Three tests had sufficient data to undertake meta-analysis: Crystal VC, Cholera Screen, and IP dipstick. For Crystal VC separate meta-analyses were carried out for samples tested directly (“direct samples”) and samples enriched in Alkaline Peptone Water (APW) prior to testing (“enriched samples”); for IP dipstick, only direct samples could be included in the meta-analysis, as there was insufficient comparable data on enriched samples; for Cholera Screen all samples were tested directly.

Table 2 reports a summary of results obtained from the meta-analyses and the SROC analysis.

**Table 2:** Summary of results of meta-analyses.

Test	No. Studies Included	Total Sample Size (Range)	Sensitivity (95% CI): range reported in included studies	Sensitivity Meta-estimate (95% CI)	Specificity (95% CI): range reported in included studies	Specificity Meta-estimate (95% CI)	Area Under Curve (AUC) estimate
Crystal VC–Direct Samples	8 [10, 32, 34, 40, 45, 47, 50, 56]	7243 (76–5865)	65.6 (52.7–77.1) to 100.0 (82.4–100.0)	93.3 (83.7–97.5)	60.0 (53.3–66.5) to 91.8 (81.9–97.3)	77.7 (70.7–83.3)	0.865
Crystal VC–Enriched Samples	5 [10, 24, 30, 32, 40]	1614 (100–673)	68.3 (51.9–81.9) to 98.9 (94.1–100.0)	85.5 (68.1–94.4)	90.0 (55.5–99.7) to 100.0 (94.4–100.0)	98.3 (92.8–99.6)	0.848
Cholera Screen–Direct Samples	3^1^ [25, 29, 39]	250 (17–99)	98.0 (93.3–100.0) to 100.0 (69.2–100.0)	98.6 (94.7–99.7)	22.2 (6.4–47.6) to 100.0 (93.3–100.0)	78.1 (18.9–98.2)	0.966
IP dipstick–Direct Samples	2^2^ [49, 59]	414 (102–172)	93.1 (84.5–97.7) to 98.5 (91.8–100.0)	95.3 (91.1–97.5)	77.0 (67.5–84.8) to 95.9 (88.6–99.2)	87.4 (73.6–94.5)	0.969

^1^One study undertaken in 2 separate locations so 4 results included

^2^One study undertaken in 2 separate locations so 3 results included

For tests on direct samples, Cholera Screen showed the highest sensitivity meta-estimate of the analysed tests, at 98.6% (95% CI: 94.7–99.7), with the lowest sensitivity meta-estimate reported in Crystal VC. Similarly, the latter test also had the lowest specificity meta-estimate, reported at 77.7% (CI 70.7–83.3), with the highest noted for the IP dipstick test.

Relating to tests used on enriched samples, only data from the Crystal VC is available. Considered alongside the other tests, the sensitivity meta-estimate is the lowest overall at 85.5% (68.1–94.4), however the specificity meta-estimate is highest overall at 98.3% (92.8–99.6).

Heterogeneity in sensitivity and specificity was seen across studies for each of the four meta-analyses, as can be seen from the ranges reported in Table 2. The highest variation was seen in the specificity of the Cholera Screen test, where the lowest reported specificity was 22.2% (6.4–47.6%) [25] and the highest 100.0% (93.3–100.0%) [39]. Forest plots providing a visual representation of the variation in sensitivity and specificity across tests and studies can be found in S3 Appendix.

The area under the curve estimates are high (greater than 0.8) across all tests, with the IP dipstick showing the highest AUC of 0.969. SROC curves plotting the sensitivity against false positive rate are reported in S3 Appendix.

### Results: Narrative synthesis of sensitivity and specificity

Given meta-analysis was not possible for the majority of tests, a narrative synthesis was also undertaken to capture diagnostic accuracy results. Tests were split into three categories: immunologically-based tests, PCR-based tests, and other test-types. We present the findings of the narrative analysis in the forthcoming section.

Table 3 presents a summary of findings of the diagnostic accuracy for each test. A full breakdown of the sensitivity and specificity results of individual studies can be found in S4 Appendix. Additionally, a full dataset of extracted and back-calculated values of sensitivity, specificity, true positives, false positives, true negatives and false negatives for each study can be found in S5 Appendix.

**Table 3:** Summary of findings: Sensitivity and specificity.

Test	Intended location	Diagnostic target—as reported in studies	Sample type	Enrich-ment step?	Reference	No. Studies	Study IDs	Sensitivity % (95% CI)	Specificity % (95% CI)
**Immunologically-based tests detecting lipopolysaccharides or proteins**									
Artron RDT	Field	Antigens of *V*. *cholerae* O1 and O139	Stool	No	Culture	1	Matias 2017 [45]	*98*.*6 (92*.*7–100)*	*69*.*1 (55*.*2–80*.*9)*
Bead ELISA	Laboratory	Cholera toxin	Stool	No	Culture	3	**Range of point estimates**	**69.9–84.7**	**63.6–81.8**
							Ramamurthy 1996 [55] (mAb-based)	*69*.*9 (57*.*9–79*.*8)*^1^	*81*.*8 (59–94)*^1^
							Ramamurthy 1996 [55] (pAb-based)	*82*.*1 (71*.*1–89*.*8)*^1^	*63*.*6 (40*.*8–82)*^1^
							Ramamurthy 1993 [54]	*82*.*9 (71*.*6–90*.*5)*^1^	*79*.*2 (65*.*5–88*.*7)*^1^
							Ramamurthy 1992 [53]	*84*.*7 (72*.*5–92*.*5)*^1^	*81*.*3 (53*.*7–95)*^1^
BengalScreen	Field	Lipopolysaccharide antigen of *V*. *cholerae* O139	Stool	No	Culture	1	Hasan 1995 [37]	*95 (71*.*9–99*.*7)*^1^	*100 (75*.*9–100)*^1^
Bengal DFA	Field	Lipopolysaccharide antigen of *V*. *cholerae* O139	Stool	No	Culture	1	Hasan 1995 [37]	*100 (79*.*1–100)*^1^	*100 (75*.*9–100)*^1^
Bengal SMART	Field	’A’ factor of *V*. *cholerae* O139	Stool	No	Culture	1	Qadri 1995 [52]	*97 (89*.*5–99*.*1)*^1^	*100 (95*.*5–100)*^1^
Cholera Screen	Field	’A’ factor of *V*. *cholerae* lipopolysaccharide O1	Stool	No	Culture	3	**Range of point estimates**	**98–100**	**22.2–100**
							Colwell 1992 [29] (Guatemala)	*100 (65*.*5–100)*^1^	42.9 (11.8–79.8)^1^
							Colwell 1992 [29] (Bangladesh)	*98 (88–99*.*9)*^1^	77.8 (57.3–90.6)^1^
							Carillo 1994 [25]	*98*.*8 (92*.*4–99*.*9)*^1^	22.2 (7.4–48.1)^1^
							Islam 1994 [39]	*100 (39*.*58–100)*^1^	100 (91.58–100)^1^
Cholkit	Field	Lipopolysaccharide antigen of *V*. *cholerae* O1	Stool	No	Culture	2	**Range of point estimates**	**79.4–97.7**	**87.4–96.5**
							Islam 2019 [40]	*79*.*4 (62*.*1–91*.*3)*	*87*.*4 (85*.*5–89*.*1)*
							Sayeed 2018 [56]	*97*.*7 (88*.*4–99*.*9)*^2^	*96*.*5 (88*.*6–99*.*6)*^2^
		Lipopolysaccharide antigen of *V*. *cholerae* O1	Stool	Yes	Culture	1	Islam 2019 [40]	*66*.*7 (47*.*2–82*.*7)*	*94*.*4 (91*.*7–96*.*5)*
Co-agglutination test (COAT)	Field	Lipopolysaccharide antigens of *V*. *cholerae* O139	Stool	No	Culture	1	Qadri 1994 [51]	*92 (84*.*1–96*.*5)*^1^	*100 (96*.*7–100)*^1^
Cholera DFA	Laboratory	’A’ factor of *V*. *cholerae* lipopolysaccharide O1	Stool	No	Culture	1	Hasan 1994a [35]	*100 (82*.*2–100)*^1^	*100 (80*.*8–100)*^1^
Cholera diagnostic kit (mAb-based dot-blot ELISA)	Field	*V cholerae* O1 antigen A	Stool	Yes	Culture	1	**Range of point estimates**	**95.2–100**	**100**
							Supawat 1994 [57] (patients)	*100 (73*.*2–100)*^1^	*100 (97*.*6–100)*^1^
							Supawat 1994 [57] (household contacts)	*95*.*2 (74*.*1–99*.*8)*^1^	*100 (98*.*8–100)*^1^
Cholera SMART	Field	Antigen A of *V*. *cholerae* O1 lipopolysaccharide	Stool	No	Culture	3	**Range of point estimates**	**58–100**	**95–100**
							Bolaños 2004 [23]	*100 (74*.*7–100)*^1^	*100 (71*.*7–100)*^1^
							Hasan 1994b [36] (Bangladesh)	*95*.*6 (76–99*.*8)*^1^	*100 (80*.*8–100)*^1^
							Kalluri 2006 [42] (field techs)	*58 (46–71)*	*95 (91–98)*
							Kalluri 2006 [42] (lab techs)	*83 (75–90)*	*88 (82–93)*
		Antigen A of *V*. *cholerae* O1 lipopolysaccharide	Stool	No	Cholera Screen	1	Hasan 1994b [36] (Mexico)	*100 (90*.*2–100)*^1^	*100 (92*.*8–100)*^1^
Crystal VC	Field	Lipopolysaccharide antigen of *V*. *cholerae* O1 and O139	Stool	No	Bacterial Culture	7	**Range of point estimates**	**65.6–98.6**	**49.2–98.4**
							George 2014 [32]	*65*.*6 (52*.*7–77*.*1)*	*91*.*8 (81*.*9–97*.*3)*
							Page 2012 [50] (clinicians)	*93*.*8 (89*.*2–97*.*2)*^2^	*78*.*4 (59*.*6–98*.*7)*^2^
							Page 2012 [50] (lab techs)	*93*.*0 (88*.*3–96*.*6)*^1^	*85*.*2 (69*.*8–99*.*2)*^1^
							Ley 2012 [43]	*93*.*1 (88*.*7–96*.*2)*^2^	*49*.*2 (44*.*3–54*.*1)*^2^
							Mukherjee 2010 [47]	*91*.*70 (95% CI not available)*	*72*.*90 (95% CI not available)*
							Islam 2019 [40]	*72*.*2 (64*.*6–78*.*9)*	*77*.*1 (75*.*9–78*.*2)*
							Sayeed 2018 [56]	*97*.*5 (87*.*5–99*.*9)*^2^	*98*.*4 (92*.*0–99*.*9)*^2^
							Matias 2017 [45]	*98*.*6 (96*.*5–99*.*6)*	*71*.*1 (64*.*6–76*.*9)*
		Lipopolysaccharide antigen of *V*. *cholerae* O1 and O139	Stool	Yes	Bacterial Culture	3	**Range of point estimates**	**75–98.9**	**90–98.4**
							George 2014 [32]	*75 (62*.*6–85)*	*98*.*4 (91*.*2–100)*
							Bwire 2017 [24]	*98*.*9 (94*.*09–99*.*97)*	*90 (55*.*5–99*.*75)*
							Islam 2019 [40]	*68*.*3 (51*.*9–81*.*9)*;	*90*.*8 (88*.*1–92*.*9)*
		Lipopolysaccharide antigen of *V*. *cholerae* O1 and O139	Stool	No	PCR	2	**Range of point estimates**	**94.4–97**	**75–79.7**
							Ontweka 2016 [10]	*94*.*4 (81*.*3–99*.*3)*	*79*.*7 (67*.*8–88*.*7)*
							Harris 2009 [34]	*97 (88*.*7–99*.*5)*^1^	*75 (56*.*2–87*.*9)*^1^
		Lipopolysaccharide antigen of V. cholerae O1 and O139	Stool	Yes	PCR	2	**Range of point estimates**	**86.1–89.3**	**98.9–100**
							Ontweka 2016 [10]	*86*.*1 (70*.*5–95*.*3)*	*100 (94*.*4–100)*
							Debes 2016 [30]	*89*.*3 (71*.*8–97*.*7)*^1^	*98*.*9 (97*.*8–99*.*6)*^1^
		Lipopolysaccharide antigen of *V*. *cholerae* O1 and O139	Stool	No	Culture or PCR	1	**Range of point estimates**	**88.2–91.9**	**82.6–88.6**
							Page 2012 [50] (clinicians)	*91*.*9 (87–95*.*4)*	*82*.*6 (71*.*6–90*.*7)*
							Page 2012 [50] (lab techs)	*88*.*2 (82*.*6–92*.*4)*	*88*.*6 (78*.*7–94*.*9)*
		Lipopolysaccharide antigen of *V*. *cholerae* O1 and O139	Water	Yes	Culture	2	**Range of point estimates**	**65.6–87**	**99.6–100**
							Rashid 2017 [1]	*65*.*6 (55*.*2–75)*	*99*.*6 (99*.*2–99*.*9)*
							Chakraborty 2013 [28]	*87 (74*.*9–94*.*3)*^1^	*100 (99–100)* ^1^
Dot-blot ELISA	Laboratory	*V*. *cholerae* O1 antigen	Stool	No	Culture	1	Chaicumpa 1995 [26]	*63 (53*.*1–71*.*3)*^1^	*97 (82–99*.*8)*^1^
		*V*. *cholerae* O139 antigen	Stool	Yes	Culture	1	Chaicumpa 1998 [27]	*100 (89*.*6–100)*^1^	*99*.*95 (99*.*8–99*.*9)*^1^
IP dipstick	Field	*V*. *cholerae* O1 lipopolysaccharide	Stool	No	Culture	3	**Range of point estimates**	**93–98.5**	**67–95.9**
							Wang 2006 [59]	*93 (87–99)*	*77 (69–85)*
							Kalluri 2006 [42] (field techs);	*93 (87–97)*	*67 (60–74)*
							Kalluri 2006 [42] (lab techs)	*94 (88–98)*	*76 (70–82)*
							Nato 2003 [49] (Madagascar)	*98*.*5 (90*.*7–99*.*9)*^1^	*95*.*9 (87*.*8–98*.*9)*^1^
							Nato 2003 [49] (Bangladesh)	*94*.*2 (83*.*1–98*.*5)*^1^	*84*.*0 (70*.*3–92*.*4)*^1^
		*V*. *cholerae* O1 lipopolysaccharide	Stool	Yes	Culture	2	**Range of point estimates**	**97–97**	**92.4–97**
							Wang 2006 [59]	*97 (93–100)*	*97 (95–100)*
							Bhuiyan 2003 [22]	*97 (88*.*7–99*.*5)*^1^	*92*.*4 (82*.*5–97*.*2)*^1^
		*V*. *cholerae* O139 lipopolysaccharides	Stool	No	Culture	1	Nato 2003 [49] (Bangladesh)	*100 (95–100)* ^1^	*92*.*5 (82*.*7–97*.*2)*^1^
		V. cholerae O139 lipopolysaccharides	Stool	Yes	Culture	1	Bhuiyan 2003 [22]	*92*.*6 (74*.*2–98*.*7)*^1^	*98*.*1 (92*.*8–99*.*7)*^1^
Latex agglutination test	Field	Lipopolysaccharide antigen of *V*. *cholerae* O1 and O139	Stool	No	Culture	1	Carillo 1994 [25]	*100 (94*.*4–100)*^1^	*33 (14*.*4–58*.*8)*^1^
Medicos dipstick	Field	*V cholerae* O1 (exact target and mechanism unknown)^3^	Stool	No	Culture	1	**Range of point estimates**	**84–88**	**79–80**
							Kalluri 2006 [42] (field techs)	*84 (77–91)*	*79 (73–85)*
							Kalluri 2006 [42] (lab techs)	*88 (81–94)*	*80 (73–95)*
Pathogen Detection Kit (PDK)	Field	Antigen A of *V*. *cholerae* O1 lipopolysaccharide	Stool	No	Culture	1	Bolaños 2004 [23]	*100 (71*.*7–100)*	*86 (56*.*2–97*.*5)*
SD Bioline	Field	Antigens of *V*. *cholerae* O1 and O139	Stool	No	Culture or PCR	1	Mwaba 2018 [48]	*90*.*9 (81*.*3–96*.*6)*	*95*.*2 (89*.*1–98*.*4)*
		Antigens of *V*. *cholerae* O1 and O139	Stool	Yes	Culture or PCR	1	Mwaba 2018 [48]	*95*.*5 (87*.*3–99*.*1)*	*100 (96*.*5–100)*
		Antigens of *V*. *cholerae* O1 and O139	Stool	No	Culture	1	Matias 2017 [45]	*81*.*1 (75*.*6–85*.*8)*	*92*.*8 (88*.*4–95*.*9)*
Two-tip dipstick ELISA (sandwich ELISA)	Field	*V*. *cholerae* O1 and O139 antigens	Stool	Yes	Culture	1	Tuteja 2007 [58]	*100 (91*.*4–100)*^1^	*100 (82*.*2–100)*^1^
		*V*. *cholerae* O1 and O139 antigens	Water	Yes	Culture	1	Tuteja 2007 [58]	*100 (31–100)* ^1^	*100 (90*.*6–100)*^1^
Vch-UPT-LF	Field	*V*. *cholerae* O1 antigen	Water	No	Combination (culture or 2+ of colloidal gold assay, real time fluorescent PCR, Vch-UPT-LF)	1	Hao 2017 [33]	*100 (71*.*7–100)*	*100 (94*.*5–100)*
		Vibrio cholerae O139 antigen	Water	No	As above	1	Hao 2017 [33]	*100 (19*.*8–100)*	*99 (93*.*4–99*.*9)*
**PCR-based tests detecting genes or nucleic acids**									
Multiplex PCR (with: O139-rfb primers—O139F-2, O139R-2; O1-rfb primers—O1F-2, O1R-2; cholera toxin primers—VCT1, VCT2)	Laboratory	*V*. *cholerae* O1 and O139 rbf-specific genes and the ctxA gene.	Stool	No	Culture	1	Sayeed 2018 [56]	*73*.*6 (58*.*5–85*.*7)*^2^	*97*.*2 (93*.*2–99*.*2)* ^2^
		*V*. *cholerae* O1 and O139 rbf-specific genes and the ctxA gene.	Stool	Yes	Culture	1	Hoshino 1998 [38]	*100 (88*.*6–100)*^1^	*95 (87*.*5–98*.*4)*^1^
PCR (with primers specific to espM gene)	Laboratory	epsM gene of *V*. *cholerae*	Water	Yes	Culture	1	Momtaz 2013 [46]	*100 (31–100)* ^1^	*100 (98*.*9–100)*^1^
PCR (with primers specific to *V*. *cholerae* O1)	Laboratory	Cholera Toxin gene of *V*. *cholerae* O1.	Stool	Yes	Culture	1	Ramamurthy 1993 [54]	*100 (93*.*5–100)*^1^	*55 (40*.*6–68*.*2)*^1^
PCR assay (with new primers O139-1 and O139-2)	Laboratory	Chromosomal region of *V*. *cholerae* O139	Stool	No	Culture	1	Albert 1997 [21]	*94 (84*.*7–98*.*1)*^1^	*100 (95*.*9–100)*^1^
TaqMan Array Card (singleplex real time PCR format)	Laboratory	toxR gene	Stool	No	Conventional assay	1	Liu 2013 [44]	*100 (59*.*8–100)*^1^	*100 (93*.*7–100)*^1^
		toxR gene	Stool	No	PCR Luminex	1	Liu 2013 [44]	*100 (62*.*9–100)*^1^	*100 (95*.*4–100)*^1^
**Other test types: real-time cell analysis, and selective media-based tests**									
Cholera toxin real-time cell analysis	Laboratory	Cholera toxin	Stool	No	Combination (2+ matching results among real-time PCR, VET-RPLA, and CT-RTCA)	1	Jin 2013 [41]	*90 (72*.*3–97*.*4)*^1^	*97 (89*.*1–99*.*5)*^1^
ChromID Vibrio	Laboratory	*V*. *cholerae* bacterial strains	Stool	No	Combination (by Vitek 2 and/or ID 32 E biochemical strips, PCR, or comparison of ChromID Vibrio with TCBS)	1	Eddabra 2011 [31]	*79 (48*.*8–94*.*3)* ^1^	*100 (75*.*9–100)* ^1^
		*V*. *cholerae* bacterial strains	Stool	Yes	As above	1	Eddabra 2011 [31]	*100 (73*.*2–100)*^1^	*100 (75*.*9–100)*^1^
TCBS	Laboratory	*V*. *cholerae* bacterial strains	Stool	No	As above	1	Eddabra 2011 [31]	*79 (48*.*8–94*.*3)*^1^	*50 (25*.*5–74*.*5)*^1^
		*V*. *cholerae* bacterial strains	Stool	Yes	As above	1	Eddabra 2011 [31]	*100 (73*.*2–100)*^1^	*50 (25*.*5–74*.*5)*^1^

^1^Confidence Intervals calculated from raw numbers provided in paper

^2^Estimate calculated using Bayesian latent class modelling

^3^Exact target and mechanism unknown, assumed immunologically-based

#### Immunologically-based tests detecting lipopolysaccharides or proteins

Twenty immunologically-based tests, with a total of 35 sub-groups, were included in the narrative synthesis.

*Sensitivity*. Of those tests intended for field use, the most frequently studied test–the Crystal VC–had reported sensitivities ranging from 65.6% (testing directly on stool samples with bacterial culture reference [1]) to 98.9% (on enriched stool samples with bacterial culture reference [24]). Several tests had reported sensitivity of 100% (nine tests, 12 sub-groups): the Cholera SMART, Cholera Screen, IP dipstick, two-tip dipstick ELISA, Vch-UPT-LF, Cholera diagnostic kit, Pathogen Detection Kit (PDK), Bengal DFA, and latex agglutination tests. Studies assessing these tests included those using both stool and water samples, enriched and non-enriched samples, and culture and combination reference tests. Notably, the majority of these tests were assessed in only one study, thus little can be inferred in relation to the high sensitivity. However, three tests—Cholera SMART, the IP dipstick, and Cholera Screen–were assessed by more than one study, and a broader range of sensitivities was found. While a sensitivity of 100% was reported for Cholera SMART in Bolaños 2004 [23] and Hasan 1994b [36], Kalluri 2006 [42] reported a sensitivity of only 58% when the test was undertaken by field technicians (83% when undertaken by laboratory technicians). Additionally, 30% of samples were marked ‘indeterminate’ by field technicians. While Nato 2003 [49] found a sensitivity of 100% for the IP dipstick, when targeting the O139 lipopolysaccharide on directly tested samples, 92.6% was reported by Bhuiyan 2003 [22] when samples were enriched. Additionally, when the O1 lipopolysaccharide was targeted, a range of 93–98.5% was reported in three studies on direct samples [42, 49, 59] and 97% in two studies on enriched samples [22, 59].

Of those tests intended for laboratory use, the bead ELISA and dot-blot ELISA were reported in more than one study, although on different samples from the same study groups. The highest sensitivities were found for the Cholera DFA [35] and dot-blot ELISA [27]. Chaicumpa 1998 [27] reported higher sensitivities for the dot-blot ELISA when stool samples were enriched versus directly tested (100% compared to 63%).

*Specificity*. In field-based tests 100% specificity was reported in 14 sub-groups for 11 tests. Of these, only Crystal VC was found to have specificity of 100% by more than one study. Ontweka 2016 [10] reported 100% (95% CI: 94.4–100%) specificity in enriched stool samples with a PCR reference, and Chakraborty 2013 [28] (100% specificity, 95% CI 99–100%) in enriched water samples with a culture reference.

Additionally, some tests had 100% specificities reported by one study, with much lower specificities reported by others: whilst one study (Islam 1994 [39]) reported specificity of 100% in the Cholera Screen test, notably low specificities were reported by two other studies: 42.9% (95% CI 11.8–79.8%) in Guatemala in Colwell 1992 [29], and 22.2% (95% CI 7.4–48.1%) in Carillo 1994 [25].

One laboratory-based test reported specificity of 100%: the Cholera DFA (Hasan 1994a [35]).

A number of studies reported tests with both 100% sensitivity and specificity: Cholera SMART (when direct stool samples tested with Cholera Screen reference, in Hasan 1994b [36]); the two-tip dipstick ELISA (both enriched and direct stool samples, in Tuteja 2007 [58]), Cholera Screen (on direct stool samples with bacterial culture reference, in Islam 1994 [39]), the Vch-UPT-LF (when targeting the O1 antigen, Hao 2017 [33]), the Bengal DFA (Hasan 1995 [37]), and the Cholera DFA (Hasan 1994a [35]). However, these results where only found in single studies.

#### PCR-based tests

All but one of the five PCR tests were assessed by only one study, which limits interpretation. However, four of five studies reported sensitivity of 100%. In Albert 1997 [21], the PCR assay with new primers O139-1 and O139-2 had a sensitivity of 94%. In the Multiplex PCR, where two studies reported results, Hoshino 1998 [38] reported sensitivity of 100% on enriched samples, whereas Sayeed 2018 [56] reported sensitivity of 73.6% on direct samples (where sensitivity was estimated using Bayesian latent class modelling).

Three of the five tests had specificity of 100%, with the remaining two reporting specificities of 55% (PCR with unspecified primers specific to *V*. *cholerae* [54]), and 95–97.2% (Multiplex PCR, enriched and direct samples respectively [38, 56]).

The TaqMan Array Card was assessed against both a conventional assay and PCR Luminex reference, and was found to have sensitivity and specificity of 100% in both instances [44].

#### Other test types: Selective media-based tests and real-time cell analysis

One study investigated selective media-based tests–the ChromID Vibrio, and Thiosulfate-Citrate-Bile Salts-Sucrose (TCBS) agar [31]. In both tests, direct stool samples had lower sensitivities than enriched samples (79% versus 100%, respectively). ChromID Vibrio appeared to be more specific than TCBS for both enriched and directly tested samples (100% versus 50%).

One study reported on a cholera-toxin real-time cell analysis, reporting sensitivity and specificity of 90% and 100%, respectively [41].

### Results: Other characteristics of included tests

#### Price

Price was not well reported across diagnostic tests, with information only available for four of 21 immunologically-based tests and one of five PCR-based tests. Price was reported by multiple papers for Crystal VC, and one paper each for Cholera SMART, the Medicos dipstick, and the SD Bioline. Of these, Crystal VC was the cheapest at approximately USD $1.90, per test [1, 32, 34, 43] and Cholera SMART the most expensive at USD $14 per test [42]. In contrast, the one PCR test with price available was considerably more expensive: the TaqMan Array Card was USD $60 per card [44]. However, it was unclear from the text how many samples a single TaqMan Array Card could process.

#### Test time

Test time was similarly more comprehensively reported across immunologically-based tests than PCR-based tests. Immunologically-based tests ranged in testing time from two minutes (Pathogen Detection Kit [23]) or less than five minutes (Cholera Screen [29, 39] and BengalScreen [37]) to 3.5 hours (Bead ELISA [53]). Tests intended for field use reported times between two minutes and less than two hours [23, 57], and tests intended for laboratory detection reported times from less than 30 minutes to 3.5 hours [35, 53]. The two-minute estimate for the Pathogen Detection Kit was for samples that were clinically deemed to have a high probability of cholera [23].

PCR-based tests generally did not specify turnaround time, with the exception of the Multiplex PCR [38] which took approximately five hours.

#### Ease of use and training

No information on ease of use or training was provided for PCR-based tests, however given these were all laboratory-based we can assume technical skill was required. Multiple studies reported ease of use for a number of different immunologically-based tests, however few specifics were given beyond ‘simple’ or ‘easy to use’, or ‘easy to perform’ [29, 37]. The exception to this was Kalluri 2006 [42] reporting on the Cholera SMART: while Hasan 1994b [36] described the test as simple and easy to use, users in Kalluri 2006 [42] reported that “the SMART device was often difficult to interpret and was frustrating to use”.

#### Other test features—Storage, internal quality control, result capturing

Where reported, immunologically-based tests displayed results as coloured lines or spots on the device [39, 43, 45, 52], and PCR-based tests as bands on a gel electrophoresis or plate [21, 38]. All studies including information about quality control included some sort of positive and/or negative control included in the test. Information on storage was not well reported, with information only available for six tests.

Further details regarding other characteristics of included tests can be found in Table 4.

**Table 4:** Other test characteristics.

Test Name	Intended location of test	Developer	Refer-ences	Test time (excl. any sample enrichment)	Price	Internal quality control	Result capturing	Notes on ease of use, training, maintenance etc.	Storage
**Immunologically-based tests detecting lipopolysaccharides or proteins**									
Artron RDT	Field	Artron Laboratories Inc, Canada	[45]	15 minutes	Not specified	Control line	Three lines—two test and a control	Not specified	Not specified
Bead ELISA	Laboratory	Not specified	[53–55]	Assay can be performed in 3.5 hours	Not specified	Control included	Not specified	Described as “easy to perform” [53]	Not specified
BengalScreen	Field	New Horizons Diagnostics	[37]	<5 minutes	Not specified	Positive and negative controls included	Visible agglutination in test circle	Described as simple and easy to use with no need for trained personnel	Not specified
Bengal DFA	Field	New Horizons Diagnostics	[37]	<20 minutes	Not specified	Positive and negative controls included	Not specified	Not specified	Not specified
Bengal SMART	Field	New Horizons Diagnostics	[52]	<15 minutes	Not specified	Not specified	Appearance of coloured spots	Not specified	Does not require refrigeration
Cholera Screen	Field	New Horizons Diagnostics Corp., Columbia, Md.	[25, 29, 40]	<5 minutes	Not specified	Positive and negative controls included	Circles marked on a slide	Described as simple [29] and with little training required [25]	Not specified
Cholkit	Field	Developed by study group	[40, 56]	15 minutes	Not yet commercially available [40]	Control line	Two lines—a test and control	Not specified	Not specified
Co-agglutination Test (COAT)	Field	Not specified	[51]	A few minutes	Not specified	Control reagent included	Two ’regions’ one control and one test region	Described as ’simple’	Not specified
Cholera DFA	Laboratory	Not specified	[35]	<30 minutes to complete staining process	Not specified	Positive and negative controls included	Appearance of yellow V. cholerae colonies	Described as ’simple’	Not specified
Cholera diagnostic kit (mAb-based dot-blot ELISA kit)	Field	Not specified	[57]	<2 hours	"Approximately one-fourth of that of the culture method"	Positive and negative control antigens included	Appearance of coloured spot indicates positive reaction	Described as “relatively simple to perform”; technicians in study received two days training.	Not specified
Cholera SMART	Field	New Horizons Diagnostics Corp., Columbia, Maryland, USA	[23, 36, 42]	10–15 minutes according to manufacturers. However, one study [42] reported mean field time as 19 minutes (range 5–40 minutes)	$14	Negative control spot	Two spots—a test and a control	Described as simple and easy to use by one study [36], conversely other users reported that "the SMART device was often difficult to interpret and was frustrating to use" [42]. No or little training required [23, 36], although offered in one study [42].	Unclear: refrigeration for long-term storage reported [42], as well as unrefrigerated storage from up to a year at room temperature [36]
Crystal VC	Field	Span Diagnostics, Surat, India (originally Institut Pasteur)	[1, 10, 24, 28, 30, 32, 34, 40, 43, 45, 47, 50, 56]	10–20 mins	$1.90 per test	Control line present	Three lines—two test lines and a control line	Described as ’simple’ and ’easy to perform’ [24, 30, 34, 43]. Half day [1, 32], to 2-day training given [10]. Others used illustrated instructions [34, 43]. Some untrained users had difficulty differentiating O1 and O139 test lines [50]. One study [50] reports that training users had no impact on test sensitivity; specificity was lower in untrained users, however difference was not statistically significant.	Stable between 4–30°C and in humid conditions.
Dot-blot ELISA	Laboratory	Not specified	[26, 27]	90 minutes [27] or between 1–3 hours [26]	‘Inexpensive’	Positive and negative controls included	Appearance of coloured spots—colour of which determined results	Described as "easy to perform"	Not specified
IP dipstick	Field	Institut Pasteur, Paris, France	[22, 42, 49, 59]	10 mins reported by manufacturer. In practice in field mean 16 min (range 3–58 mins) [42]	Not yet commercially available	Control line	Two lines—a test and control	Test requiring ’little technical experience’ [22], and lab and field technicians reported easy to use [42]. Training was reported in some studies [22, 42].	Storage at room temperature [22, 42]; stable for 21 days at 60°C, 4°C, -20°C and -80°C [49]
Latex agglutination test	Field	Denka Seiken, Tokyo, Japan	[25]	“Rapid”	Not specified	Not specified	Not specified	Reported that little training required	Not specified
Medicos dipstick	Field	Advanced Diagnostics Inc., South Plainfield, NJ, USA	[42]	10 mins reported by manufacturer. In practice mean time in field 23 mins (range 7–54 mins)	$4 per test	Not specified	Not specified	Reported easy to use and interpret; training given in study	Refrigeration for long-term storage
Pathogen Detection Kit (PDK)	Field	Intelligent Monitoring Systems, Gainsville, Florida, USA	[23]	2 mins in samples with high probability of cholera	Not specified	Not specified	Visualized on nitrocellulose membrane	Ease of use not specified, but highly qualified technical personnel not required	Not specified
SD Bioline	Field	Standard Diagnostics Inc., Korea	[45, 48]	10–20 minutes	Approx. €2 per test	Control line	Three lines—two test and a control	Matches recommendations on ease of use	Not specified
Two-tip dipstick sandwich ELISA	Field	Not specified	[58]	Not specified	Not specified	Not specified	Appearance of coloured dots	Described as simple	Not specified
Vch-UPT-LF	Field	Not specified	[33]	Not specified	Not specified	Control line present	Three lines—two test lines and a control line	Not specified	Not specified
**PCR-based tests detecting genes or nucleic acids**									
Multiplex PCR (with: O139-rfb primers—O139F-2, O139R-2; O1-rfb primers—O1F-2, O1R-2; cholera toxin primers—VCT1, VCT2)	Laboratory	Not specified	[38, 56]	~5 hours	Requires "expensive reagents" [56]	Positive and negative controls included	Bands on a plate	Requires "trained laboratory staff" [56]	Not specified
PCR (with primers specific to espM gene)	Laboratory	Not specified	[46]	"fast"	Not specified	Not specified	Not specified	Not specified	Not specified
PCR (with primers specific to V. cholera O1)	Laboratory	Not specified	[54]	Not specified	Not specified	Not specified	Not specified	Not specified	Not specified
PCR assay (with new primers O139-1 and O139-2)	Laboratory	Not specified	[21]	Not specified	Not specified	Positive and negative controls run alongside test samples	Bands on gel electrophoresis	Not specified	Not specified
TaqMan Array Card (singleplex real time PCR format)	Laboratory	Life Technologies	[44]	"Significantly shorter than that of conventional methods"	$60 for TAC	Assays spotted in duplicate and two positive controls and a negative control included	Not specified	Not specified	Not specified
**Other test types: real-time cell analysis, and selective media-based tests**									
Cholera toxin real-time cell analysis	Laboratory	RTCA system from ACEA Biosciences, San Diego, CA	[41]	Inoculation to detection time 0.89±0.51 h	Not specified	Not specified	Not specified	Not specified	Not specified
ChromID Vibrio	Laboratory	bioMérieux, Marcy l’Etoile, France	[31]	Not specified	Not specified	Not specified	Appearance of blue-green bacterial colony	Not specified	Plates stored at 4°C
TCBS medium	Laboratory	Bio-Rad (Marnes-La-Coquette, France)	[31]	Not specified	Not specified	Not specified	Appearance of blue-green bacterial colony	Not specified	Not specified

### Risk of bias and applicability

Risk of bias was assessed using the QUADAS-2 framework [19].

Sample selection was deemed low to unclear risk of bias across studies; studies assessed as unclear were graded so due to a paucity of information on selection in the record. All studies providing information on sample selection were assessed as low risk.

Risk of bias in the interpretation of the index test was assessed as high in 13 studies. In all of these, this relates to applicability concerns, as the intended location of the test did not match the location in which the study evaluated that test, as assessed by reviewers. The remaining studies were rated unclear, where information was missing, or low. Only eight studies specified that blinding was used for interpretation of test results, and one study specified that it was not used. The remaining studies were unclear.

Risk of bias in the test conduct and interpretation of the reference test was assessed as low in all but three studies. In Hao 2017 [33], Jin 2013 [41] and Eddabra 2011 [31], risk of bias was graded unclear, due to the use of complex combination references, in which two of three different test methods were required to be positive. While the remainder of the studies were graded low risk, the majority used a bacterial culture reference. The low grade was deemed appropriate as bacterial culture is considered the gold standard in cholera diagnosis; however, it has recognised limitations due to its low sensitivity (as low as 70.8% reported in Sayeed 2018 [56]). Five studies specified that reference tests were undertaken in a blinded manner, with the remaining studies being unclear.

Sample flow refers to whether samples received the same reference standard, and whether all samples were included in the analysis. While all studies used reference standards consistently across samples, three studies (Debes 2016 [30], Liu 2013 [44], Matias 2017 [45]), did not include all samples in their analysis, and did not report reasons for this exclusion.

**Fig 2:**
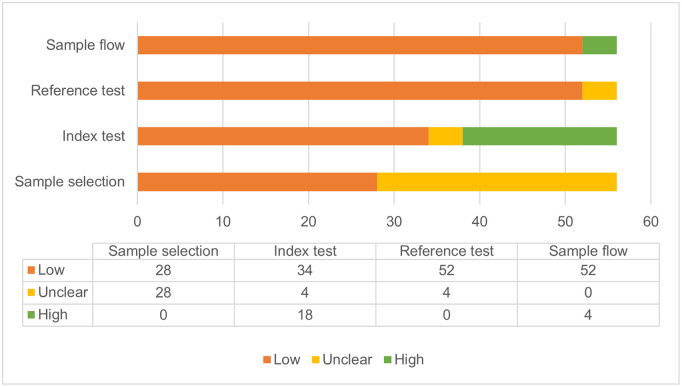
Number of studies assessed as ‘low’, ‘unclear’ and ‘high’ concern across risk of bias and applicability domains.

The results of the risk of bias and applicability assessment are shown in Fig 2. A full table of results for individual studies is available in S6 Appendix.

## Discussion

This review found 41 studies reporting on 28 different tests for cholera diagnosis and detection. The majority of these tests were immunologically-based and intended for field settings. Diagnostic accuracy of different tests appeared broadly similar, with 22 tests having both sensitivities and specificities above 95% reported by at least one study. However, accuracy was difficult to compare directly due to variations in sample handling and setting in which tests were assessed. Additionally, low sample sizes limited the validity of some assessments, particularly in those 10 studies where sample size was less than 100.

### Meta-analysis

The three tests for which meta-estimates were available (Crystal VC on direct samples and enriched samples; Cholera Screen on direct samples; and IP dipstick on direct samples), showed good diagnostic accuracy. Sensitivity meta-estimates ranged from 85.5% (68.1–94.4%) in Crystal VC on enriched samples, to 98.6% (94.7–99.7%) in the Cholera Screen test on direct samples. Specificity meta-estimates ranged from 77.7% (70.7–83.3%) in Crystal VC on direct samples, to 98.3% (92.8–99.6%) in Crystal VC on enriched samples. However, heterogeneity was seen across studies in all four meta-analyses, in particular in the sensitivities reported for Crystal VC (both on direct and enriched samples), and the specificities reported for Cholera Screen.

When interpreting sensitivity and specificity results it is critical to recognise that these are paired outcomes, which tend to be inversely correlated [60]. For this reason, statistical methods such as calculation area under the curve (AUC) and diagnostic odds ratios (DOR) are preferred, though less intuitive to interpret.

Area under the curve (AUC) estimates were all above 0.8, with two tests having estimates above 0.95 (Cholera Screen and IP dipstick), demonstrating these tests provided correct diagnoses over 95% of the time. However, the inclusion of only a small number of studies prompts caution over AUC results [61], particularly in the meta-analyses of the Cholera Screen and IP dipstick tests, where only four and three data points were included, respectively. Additionally, a high area under the curve can occur even with studies with very high specificities but low sensitivities (e.g., Carillo 1994 [25] in the Cholera Screen meta-analysis, reporting a sensitivity of only 22%).

DOR results can be found in S3 Appendix.

### Narrative synthesis: Factors affecting sensitivity and specificity

The narrative synthesis completed for all reported products indicated that multiple tests had at least one study reporting both sensitivity and specificity of over 90%. For example, for field-based tests on stool samples with no enrichment step, this included nine tests (BengalScreen, Bengal DFA, Bengal SMART, Cholera Screen, Cholkit, Cholera SMART, Crystal VC, IP dipstick and SD Bioline). However, wide variation was seen across studies, and several factors prompt caution over interpretation of sensitivity and specificity results. While bacterial culture is considered the gold standard in cholera diagnostics due to its high specificity, sensitivity is reportedly low [10, 50, 56]. This creates a situation whereby the index test may be more sensitive than the reference standard, leading to an underestimation of index test specificity; thus, accuracy of index tests assessed only against bacterial culture should be interpreted with this in mind. A couple of solutions have been used to overcome this. First, using combination references, such as culture alongside PCR [48, 50]. Second, use of Bayesian latent class analysis, which considers prior information regarding accuracy of bacterial culture (as used by Sayeed 2018 [56] and Page 2012 [50]).

The majority of tests reviewed are intended for use in the field, in cholera outbreak situations. However, the studies often assessed tests in alternate settings, such as in a lab using field samples [28, 45]. There was some evidence that studies that did assess tests in ‘real’ field settings found lower sensitivities and specificities than those using alternate settings. For example, for the Cholera SMART test, Kalluri 2006 [42] reported sensitivity of 58% during a field setting whereas Bolaños 2004 [23] reported sensitivity of 100% during laboratory assessment. However, Hasan 1994b [36], assessing the Cholera SMART in a field trial undertaken at a research centre (International Centre for Diarrhoeal Disease Research, Bangladesh) reported sensitivities of 95.6–100%. Moreover, this pattern is not seen across all tests: the Crystal VC results show no clear association between studies in contexts that match intended use, and those that do not. Ley 2012 [43] explicitly compared performance of the Crystal VC in the laboratory and the field, using the same samples, finding sensitivity and specificity of 90% and 55.6%, respectively, in the field, and 87.5% and 74.1%, respectively, in the laboratory. However, this difference was not statistically significant. Mukherjee 2010 [47] reports that the specific conditions of the field may also be impactful: during monsoon season, when *V*. *cholerae* cases were more prevalent, sensitivity and specificity of Crystal VC was 100% and 87.3%, compared to 88% and 61% during the post-monsoon and winter season when *V*. *cholerae* cases were less prevalent.

Finally, the skill level of the tester may affect test performance: Kalluri 2006 [42] found a sensitivity and specificity for Cholera SMART of 58% and 95%, respectively, when the test was undertaken by field technicians, but 83% and 88%, when undertaken by lab technicians. The study by Page 2012 [50] assessed performance of Crystal VC when undertaken by laboratory technicians and clinicians. Using Bayesian analysis, sensitivity and specificity of 93% and 85.3% was reported when undertaken by laboratory technicians, compared with 93.8% and 78.4% when undertaken by clinicians. However, these differences in specificity were not statistically significant [50].

### Narrative synthesis: Other product characteristics

Other characteristics of tests were generally poorly reported on in studies. However, a limited number of studies did report on factors such as cost, turnaround time, and ease of use–major factors which affect how tests perform in practice. Given the similarity in diagnostic accuracy results of many of the tests, it is the intended setting of a product and the product’s other technical or usability characteristics that are likely to drive decisions over which test is most appropriate in a given situation. For example, if diagnostic test cost is the ultimate factor affecting utility of a test in developing countries (as suggested by Kalluri 2006 [42]), the Crystal VC (costing $1.90 per test), or the SD Bioline (at approximately €2 per test) are the cheapest–although, any sample enrichment required will somewhat increase this cost. If turnaround time is prioritised, the Cholera Screen and the Pathogen Detection Kit have the fastest reported times, both at under 5 minutes [23, 29, 39].

Sample enrichment additionally affects turnaround time. The estimates reported in Table 4 for test time do not include enrichment time, despite numerous studies using samples enriched in Alkaline Peptone Water (APW) prior to testing [10, 24, 30, 32, 40]. Enrichment time varied from four hours in APW (Crystal VC [10]; two-tip dipstick ELISA [58]; Cholera diagnostic kit [57]; IP dipstick [22]; SD Bioline [48]) to 24 hours in APW (Crystal VC [28, 30]), which significantly increases the turnaround time of RDTs for which it may be required. Additionally, reported test-time estimates were manufacturer specified, rather than assessed by the independent evaluators. Kalluri 2006 [42] assessed the manufacturer’s specification versus actual field time taken for three tests and reported that: Cholera SMART had a 10–15 minute specification whereas actual field time ranged from five to 40 minutes (mean 19 minutes); the IP dipstick had a 10-minute specification but took between three and 58 minutes in the field (mean 16 minutes); and the Medicos dipstick had a 10 minute specification but in practice took between seven and 54 minutes (mean 23 minutes).

There was insufficient evaluation around ease of use and training requirements to draw out which tests were considered the most useable. While many studies briefly described tests as ‘simple’ or ‘easy to use’, it was not clear whether this information came from independent evaluation or manufacturer specifications. When usability was evaluated in studies, results appeared more mixed–for example with untrained users of the Crystal VC reporting difficulties differentiating O1 and O139 test lines [50], and laboratory and field technicians using Cholera SMART reporting that the device was “often difficult to interpret and was frustrating to use” [42].

### Results in the context of previous reviews

This review–using a systematic review methodology—confirms the breadth of RDTs available for cholera detection with acceptable sensitivity and specificity suggested by previous non-systematic reviews [11, 13, 14]. However, the range of scores achieved for the same tests in different studies and contexts of use reinforces concerns regarding small sample sizes raised by Dick et al. [11] and the lack of field evaluation of kits indicated by both Ramamurthy et al. [13] and Dick et al. [11].

### Limitations

To our knowledge, this is the first systematic review and meta-analysis on products for the diagnosis and detection of cholera to have been undertaken. Eleven databases of published and grey literature were searched, along with reference lists of identified studies, to capture as many relevant studies as possible. Despite this, some papers may have been missed in searches and not have been included. Additionally, given our search of predominantly English language databases, and problems accessing papers in alternate languages, our results show an Anglophone bias. As noted, data extraction from—and interpretation of–many studies was constrained by lack of detail in reports (including failure to comply with the Standards for Reporting of Diagnostic Accuracy (STARD) guidelines [62]).

## Conclusions

Our findings indicate a number of actions that would strengthen the evidence-base regarding diagnostic test accuracy for cholera using water and still samples. For example, best practice is reflected in studies that include multiple index tests on the same samples, against a reference test, rather than assessing a single index test (for example as per Sayeed 2018 [56], Matias 2017 [45], and Bolaños 2004 [23]). Additionally, given limitations with culture as a reference, combination references using culture and PCR, or alternate methods of analysis, such as Bayesian methods, which take into account prior information regarding reference test accuracy, are of particular value (as in Sayeed 2018 [56] and Page 2012 [50]).

More generally, studies need to engage much more systematically with factors that may constrain the use and accuracy of diagnostic tests in field conditions in the low-resource settings where cholera may constitute a particular risk. This includes greater emphasis on studies of sensitivity and specificity in relevant field settings (such as those by Ontweka 2016 [10], Islam 2019 [40], Page 2012 [50] and Supawat 1994 [57]); however, it is not limited to this. With limited use of RDTs for either cholera surveillance or outcome detection [15], studies of barriers to use at scale are required. In contexts of extreme poverty, where most individuals will be earning under $2.00 per day, even the tests identified here as the cheapest are likely to be experienced as unaffordable at scale. Equally, evidence of decrements in test accuracy when conducted by staff with lesser levels of training–especially when tests are presented as ‘simple to use’–points to the importance of formal usability testing [63, 64] to establish threshold competence for reliable use. Turnaround times for test results also warrant rigorous analysis given the constraints of access, transport, and communications in many low-resource settings. For point-of-care use in such settings, evidence reviewed here suggests the value of formally establishing a test maintaining attainable ‘best in class’ performance of 90%+ sensitivity and specificity with a reliable turnaround time of under 10 minutes, while making little demand of technical expertise or laboratory facilities at cost of appreciably under $2.00 per unit.

## Supporting information

S1 Appendix PRISMA-DTA checklist. (PDF)

S2 Appendix Search strategies. (PDF)

S3 Appendix Additional meta-analysis results. (PDF)

S4 Appendix Summary of findings of individual studies. (PDF)

S5 Appendix Dataset. (XLSX)

S6 Appendix Risk of bias and applicability results. (PDF)
