# Supplementary material for: Cholera diagnosis in human stool and detection in water: A systematic review and meta-analysis
Source: PLoS One. 2022 Jul 6;17(7):e0270860. doi: 10.1371/journal.pone.0270860 (PMC9258865; doi:10.1371/journal.pone.0270860)
Supplement: S2 Appendix — (PDF) [file pone.0270860.s002.pdf]

Appendix S2 – search strategies

Medline:

1. (MH "Cholera") OR "cholera" OR (MH "Cholera Toxin")
2. (AB choler* OR TI choler*)
3. 1 OR 2
4. (MH "Diarrhea") OR "diarrh#ea"
5. (MH "Water") OR "water" OR (MH "Drinking Water") OR (MH "Water Pollution") OR (MH "Water Wells") OR (MH "Water Supply") OR (MH "Water Quality") OR (MH "Waste Water") OR (MH "Water Microbiology")
6. (AB stool OR TI stool) OR (AB diarrh* OR TI diarrh*) OR (AB water OR TI water)
7. 4 OR 5 OR 6
8. (MH "Diagnosis") OR "diagnosis" OR (MH "Early Diagnosis") OR (MH "Diagnosis, Differential")
9. (MH "Limit of Detection") OR "detection"
10. (AB diagnos* OR TI diagnos*) OR (AB detect* OR TI detect*)
11. 8 OR 9 OR 10
12. 3 AND 7 AND 11
13. Limit 12 to Date of Publication: 19900101-20171231

Embase (1980 to 2017 week 40):

1. cholera/ or cholera.mp or cholera toxin/
2. choler*.ti,ab.
3. 1 or 2
4. diarrhoea/ or diarrhea.mp.
5. water quality/ or water supply/ or water.mp. or waste water/ or water pollution/ or well water/ or drinking water/ or water/ or water contamination/
6. (stool or diarrh* or water).ti,ab.
7. 4 or 5 or 6
8. diagnosis.mp. or laboratory diagnosis/ or diagnosis/ or early diagnosis/ or differential diagnosis/
9. detection.mp. or "limit of detection"/
10. (diagnos* or detect*).ti,ab.
11. 8 or 9 or 10
12. 3 and 7 and 11
13. Limit 12 to yr="1990-Current"

CINAHL:

1. (MH "Cholera") OR "cholera"
2. (AB choler* OR TI choler*)
3. 1 OR 2
4. (MH "Diarrhea") OR "diarrh#ea"
5. (MH "Feces") OR "f#eces"
6. stool
7. (MH "Water") OR "water" OR (MH "Water Pollution") OR (MH "Water Supply") OR (MH "Water Microbiology")

8. (AB stool OR TI stool) OR (AB fec* OR TI fec*) OR (AB faec* OR TI faec*) OR (AB diarrh* OR TI diarrh*) OR (AB water OR TI water)
9. 4 OR 5 OR 6 OR 7 OR 8
10. (MH "Diagnosis") OR "diagnosis" OR (MH "Early Diagnosis") OR (MH "Diagnosis, Laboratory")
11. "detection"
12. (AB diagnos* OR TI diagnos*) OR (AB detect* OR TI detect*)
13. 10 OR 11 OR 12
14. 3 AND 9 AND 13
15. Limit 13 to Date of Publication: 19900101-20171231

Scopus:

((TITLE-ABS-KEY (choler*)) AND (TITLE-ABS-KEY (diagnos* OR detect*)) AND ((TITLE-ABS-KEY (diarrh* OR water OR stool)))) AND (PUBYEAR > 1989)

Proquest:

(mesh.Exact("Cholera" OR "Cholera Toxin") OR TI,AB,IF(choler*)) AND (mesh.Exact("Diarrhea") OR TI,AB,IF(diarrh*) OR mesh.Exact("Water" OR "Water Supply" OR "Waste Water" OR "Water Wells" OR "Drinking Water" OR "Water Microbiology" OR "Water Pollution" OR "Water Quality") OR TI,AB,IF(water) OR TI,AB,IF(stool)) AND (mesh.Exact("Diagnosis" OR "Early Diagnosis" OR "Diagnosis, Differential") OR TI,AB,IF(diagnos*) OR mesh.Exact("Limit of Detection") OR TI,AB,IF(detect*)) AND pd(19900101-20171231)

Global Health Library (WHOLIS):

(mh:(cholera) OR tw:(choler*)) AND (mh:(Diarrhea) OR tw:(diarrh*) OR mh:(water) OR mh:("Water Supply") OR mh:("Waste Water") OR mh:("Water Wells") OR mh:("Drinking Water") OR mh:("Water Microbiology") OR mh:("Water Pollution") OR mh:("Water Quality") OR tw:(water) OR tw:(stool)) AND (mh:(Diagnosis) OR mh:("Early Diagnosis") OR mh:("Diagnosis, Differential") OR tw:(diagnos*) OR mh:("Limit of Detection") OR tw(detect*)) AND (year_cluster:(1990 OR 1991 OR 1992 OR 1993 OR 1994 OR 1995 OR 1996 OR 1997 OR 1998 OR 1999 OR 2000 OR 2001 OR 2002 OR 2003 OR 2004 OR 2005 OR 2006 OR 2007 OR 2008 OR 2009 OR 2010 OR 2011 OR 2012 OR 2013 OR 2014 OR 2015 OR 2016 OR 2017))

IndMed:

(cholera OR cholerae) AND (Diarrhea OR diarrhoea OR diarrhoeal OR diarrheal OR water OR stool) AND (Diagnosis OR diagnostic OR diagnose OR diagnosed OR detect OR detection OR detected)

OpenGrey:

(cholera OR cholerae) AND (Diarrhea OR diarrhoea OR diarrhoeal OR diarrheal OR water OR stool) AND (Diagnosis OR diagnostic OR diagnose OR diagnosed OR detect OR detection OR detected)

WHO IRIS:

(cholera OR cholerae) AND (Diarrhea OR diarrhoea OR diarrhoeal OR diarrheal OR water OR stool) AND (Diagnosis OR diagnostic OR diagnose OR diagnosed OR detect OR detection OR detected)

ClinicalTrials.gov:

(cholera OR cholerae) AND (Diagnosis OR diagnostic OR diagnose OR diagnosed OR detect OR detection OR detected)

ICTRP, WHO:

(Cholera AND diagnosis) OR (Cholera AND diagnostic) OR (Cholera AND diagnose) OR (Cholera AND detect) OR (Cholera AND detection)
