# Supplementary material for: Cholera diagnosis in human stool and detection in water: A systematic review and meta-analysis
Source: PLoS One. 2022 Jul 6;17(7):e0270860. doi: 10.1371/journal.pone.0270860 (PMC9258865; doi:10.1371/journal.pone.0270860)
Supplement: S3 Appendix — (PDF) [file pone.0270860.s003.pdf]

Appendix S3 – Additional meta-analysis results

Contents

Sensitivity and Specificity Forest Plots and SROC Curves 2
Crystal VC on Direct Samples 2
Crystal VC on Enriched Samples 4
Cholera Screen 6
IP Dipstick 8
DOR Analysis 10
Methods 10
Results and Discussion 11
DOR Forest Plots 12

Sensitivity and Specificity Forest Plots and SROC Curves

Crystal VC on Direct Samples

Figure 1. Forest plot results for Crystal VC - direct samples sensitivity and specificity meta-analysis

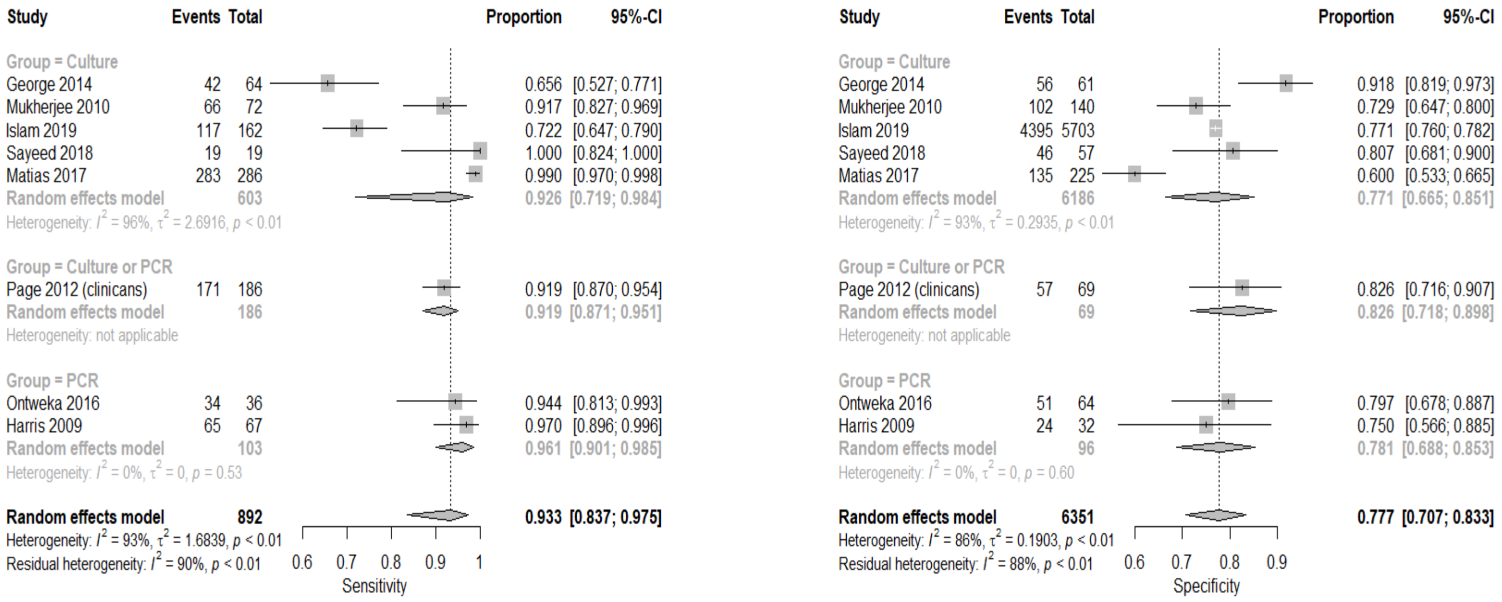

Figure 2. SROC Curve: Crystal VC - Direct Samples

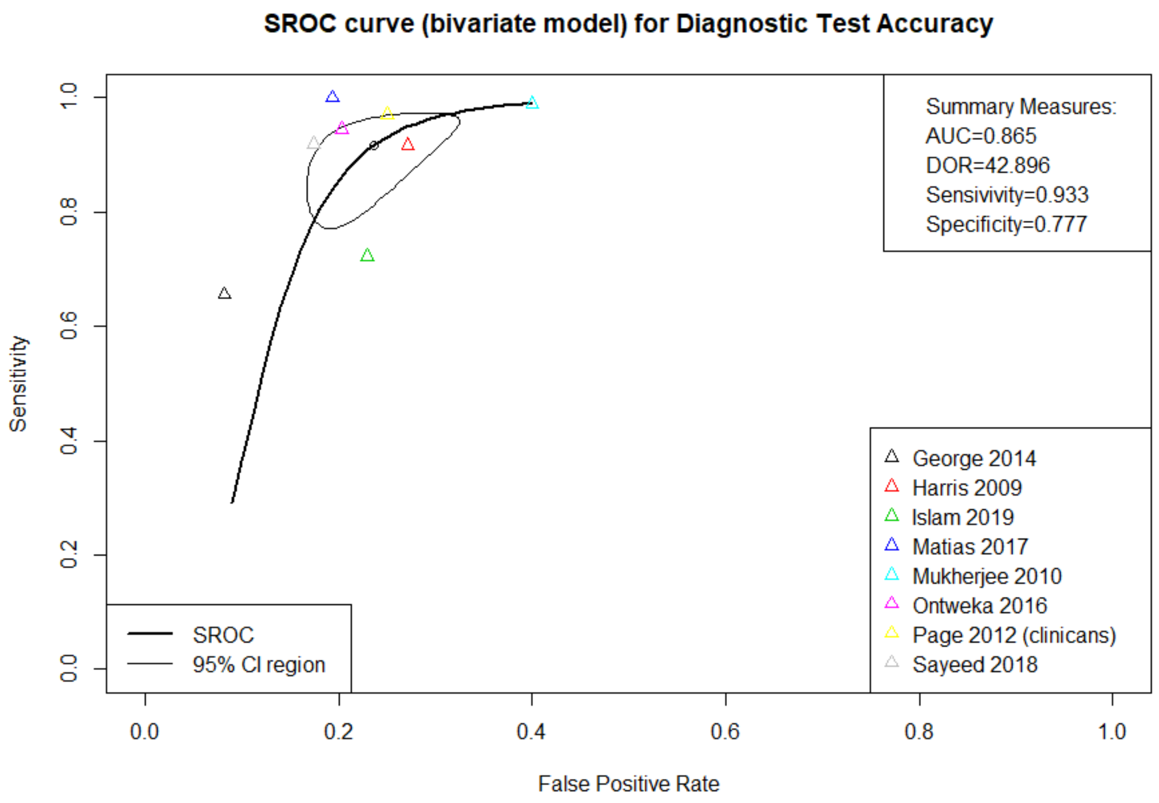

Crystal VC on Enriched Samples

Figure 3. Forest plot results for Crystal VC - enriched samples, sensitivity and specificity meta-analysis

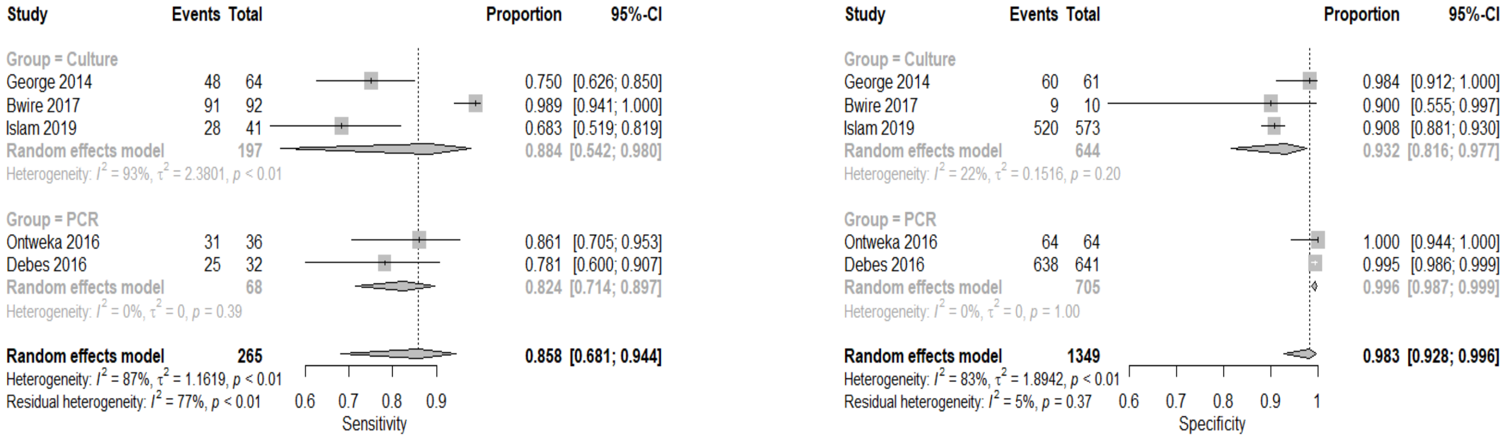

Figure 4. SROC Curve: Crystal VC – Enriched Samples

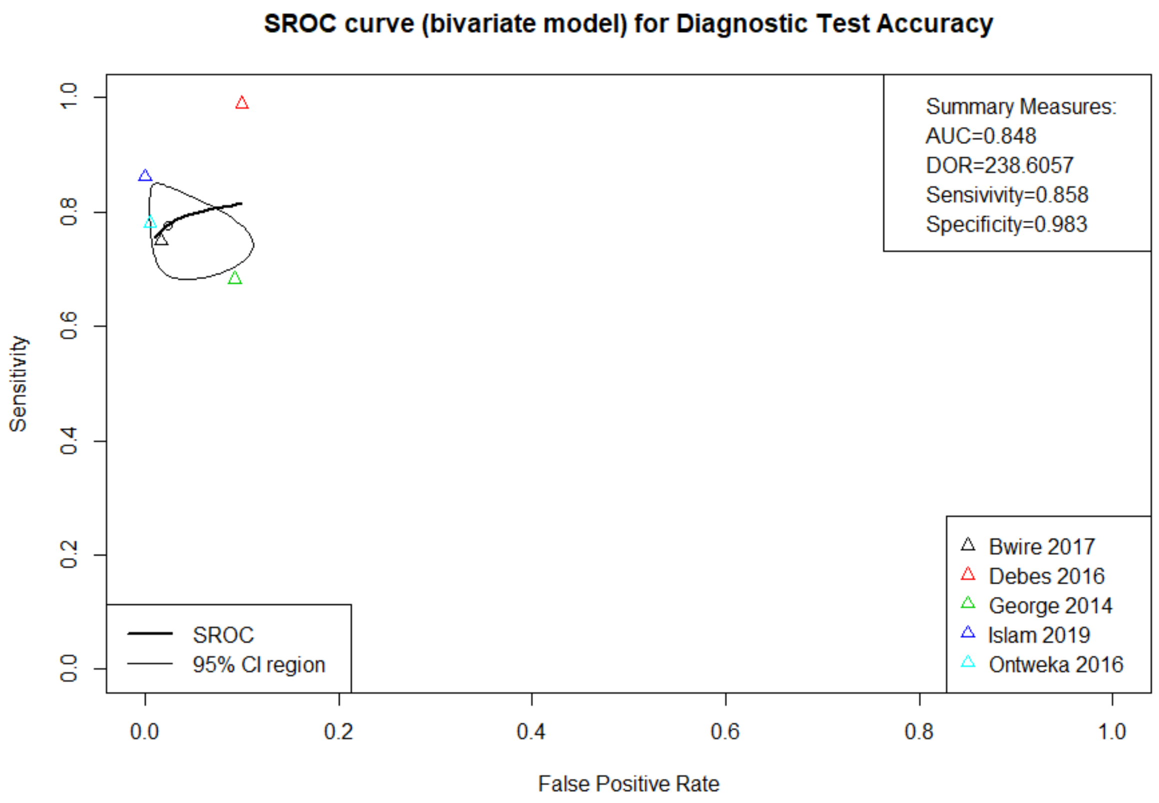

Cholera Screen

Figure 5. Forest plot results for Cholera Screen, sensitivity and specificity meta-analysis

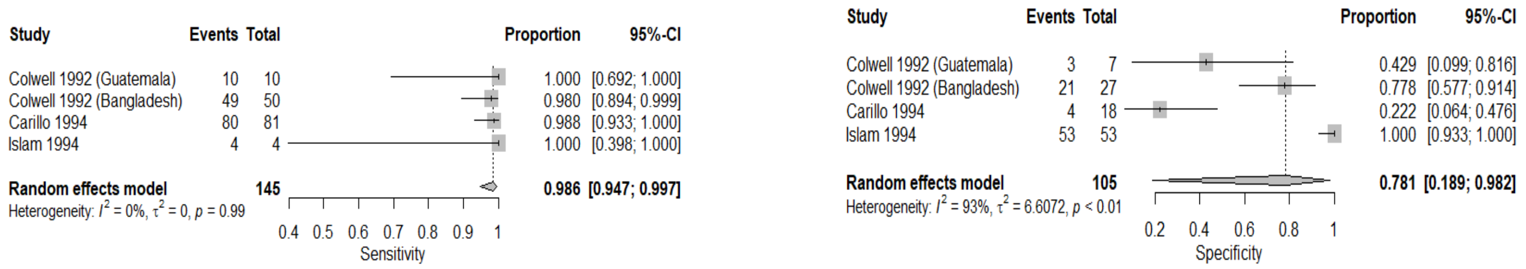

Figure 6. SROC Curve: CholeraScreen

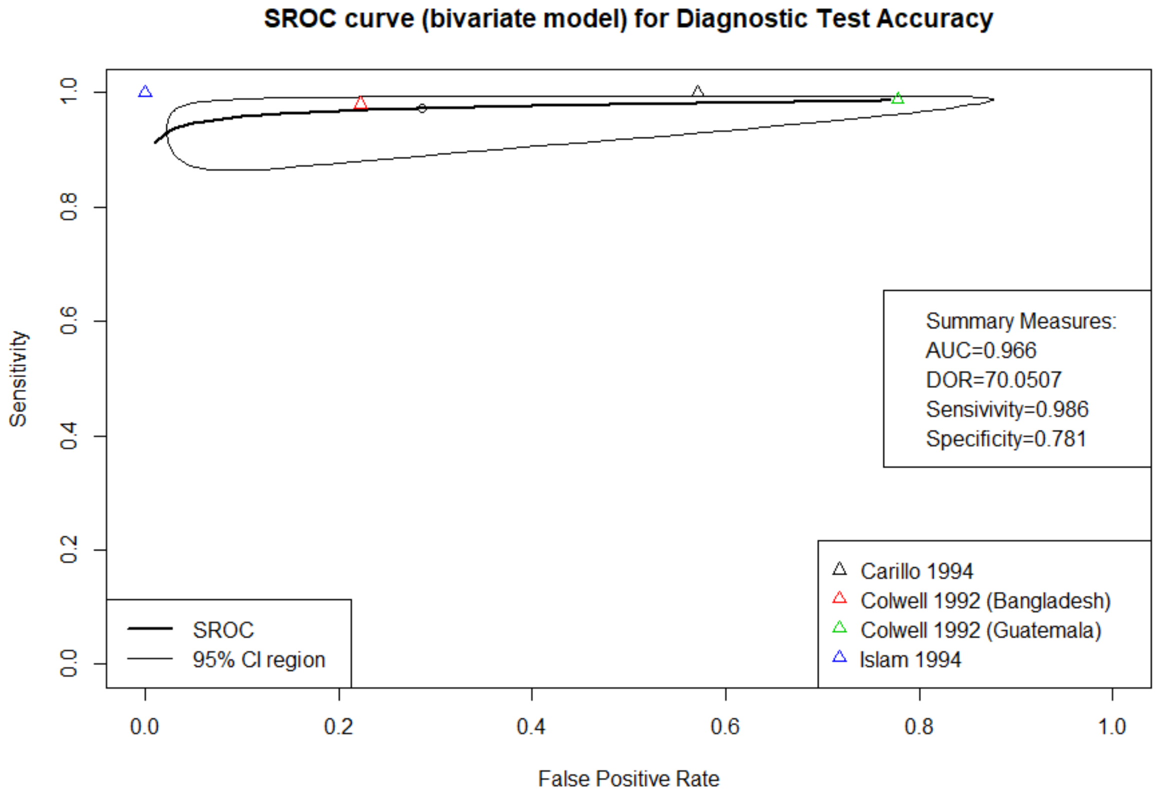

IP Dipstick

Figure 7. Forest plot results for IP dipstick, sensitivity and specificity meta-analysis

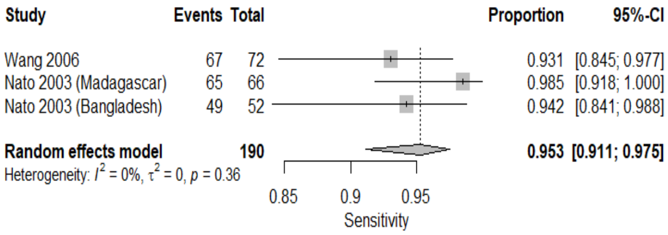

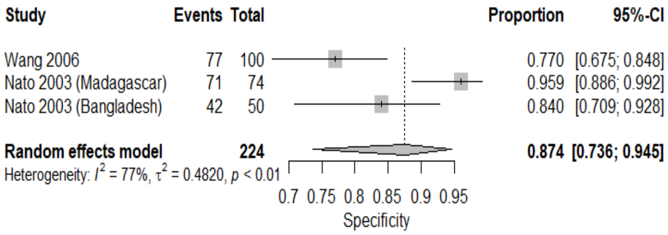

Figure 8. SROC Curve: IP Dipstick - Direct Samples

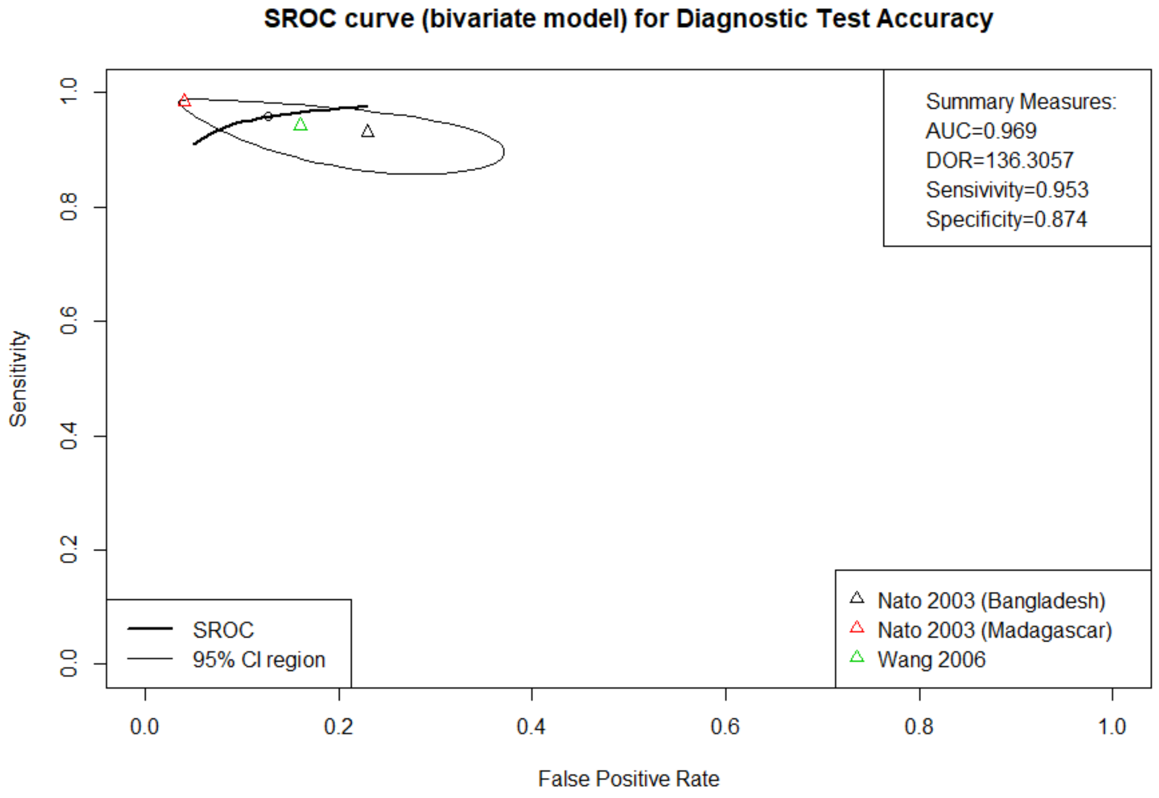

DOR Analysis

Methods

To allow comparison of tests using one measure of diagnostic accuracy, an additional analysis was undertaken on diagnostic odds ratio (DOR) according to the methods outlined in Shim et al, 2019[1]. Diagnostic odds ratio is the ratio of the odds of the index test being positive when an individual has a disease (i.e., positive result from a reference test), compared to the odds of the index test being positive when an individual does not have the disease (i.e. negative result from a reference test). A DOR of 1 therefore indicates an index test is uninformative. The DOR is calculated as follows:

$$DOR = \frac{TP \times TN}{FP \times FN}$$

Where: TP = True Positive; TN = True Negative; FP = False Positive; FN = False Negative

A random effects model was used to account for variation across studies. Only one DOR estimate per study was included in each meta-analysis to ensure no duplication of samples. Where studies had more than one estimate (e.g., due to lab technicians and field technicians both undertaking the test), priority was given to results obtained from settings most similar to that intended by the test. Multiple results from the same study group were included only if estimates were based on samples from distinct geographical locations.

[1] Shim SR, Kim S-J, Lee J. Diagnostic test accuracy: application and practice using R software. Epidemiol Health. 2019;41. doi:10.4178/epih.e2019007

Results and Discussion

Table 1. Summary of results of meta-analyses

Test	No. Studies Included [reference number]	Total Sample Size (Range)	Diagnostic Odds Ratio meta-estimate (95% CI)
Crystal VC – Direct Samples	8 [10,32,34,40,45,47,50,56]	7243 (76-5865)	42.90 (17.51-105.10)
Crystal VC – Enriched Samples	5 [10,24,30,32,40]	1614 (100-673)	238.61 (32.56-1748.70)
Cholera-Screen – Direct Samples	3[1] [25,29,39]	250 (17-99)	70.05 (14.39-340.99)
IP Dipstick – Direct Samples	2[2] [49,59]	414 (102-172)	136.31 (25.44-730.38)

[1]One study undertaken in 2 separate locations so 4 results included
[2]One study undertaken in 2 separate locations so 3 results included

The high Diagnostic Odds Ratio meta-estimates shown in Table 1 indicate that all four tests show good diagnostic accuracy: patients with a positive test have much greater odds of having cholera (as diagnosed by the reference tests) than patients with a negative test. The variability between studies within each meta-analysis is shown visually in the forest plots below.

However, these results must all be interpreted with caution. Diagnostic Odds Ratios are sensitive to studies where sensitivity or specificity are close to or at 100%, resulting in small or zero cell values during odds ratio calculation[2]. Therefore, while all four tests showed high diagnostic odds ratios, we cannot conclusively determine which test is most accurate on this basis

[2] Huang Y, Yin J, Samawi H. Methods improving the estimate of diagnostic odds ratio. Commun Stat - Simul Comput. 2018;47: 353–366. doi:10.1080/03610918.2016.1157183

DOR Forest Plots

Figure 9. Forest plot results for Crystal VC - Direct Samples, DOR meta-analysis

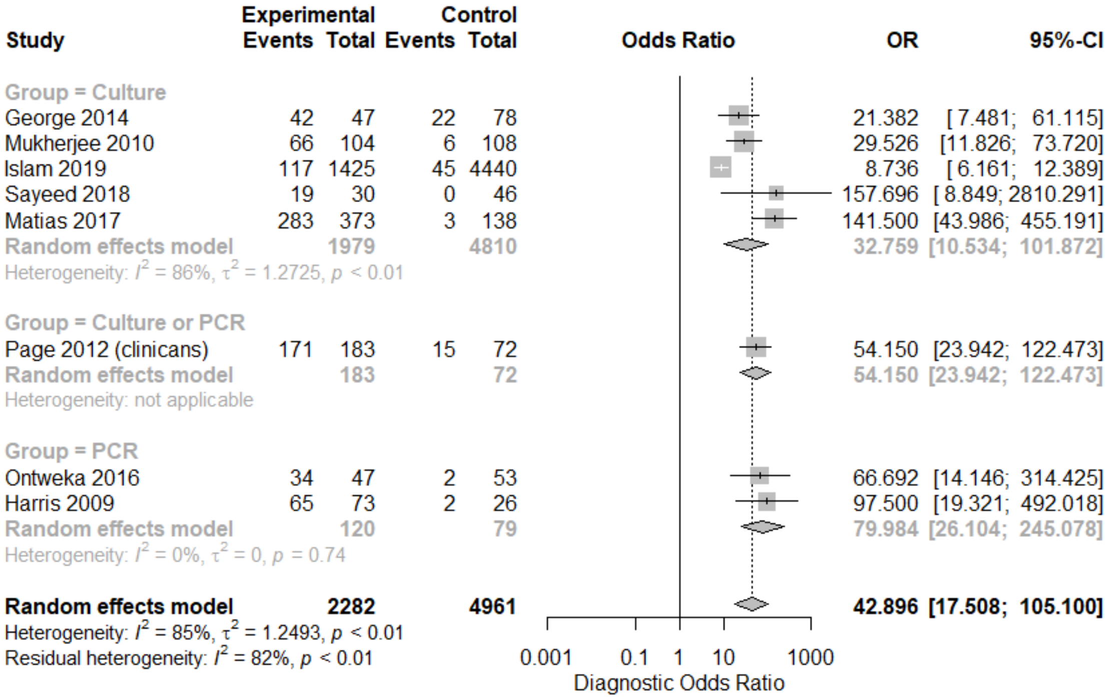

*Experimental: Events = True Positives; Total = True Positives + False Positives. Control: Events = False Negatives; Total = True Negatives + False Negatives

Figure 10. Forest plot results for Crystal VC - Enriched Samples, DOR meta-analysis

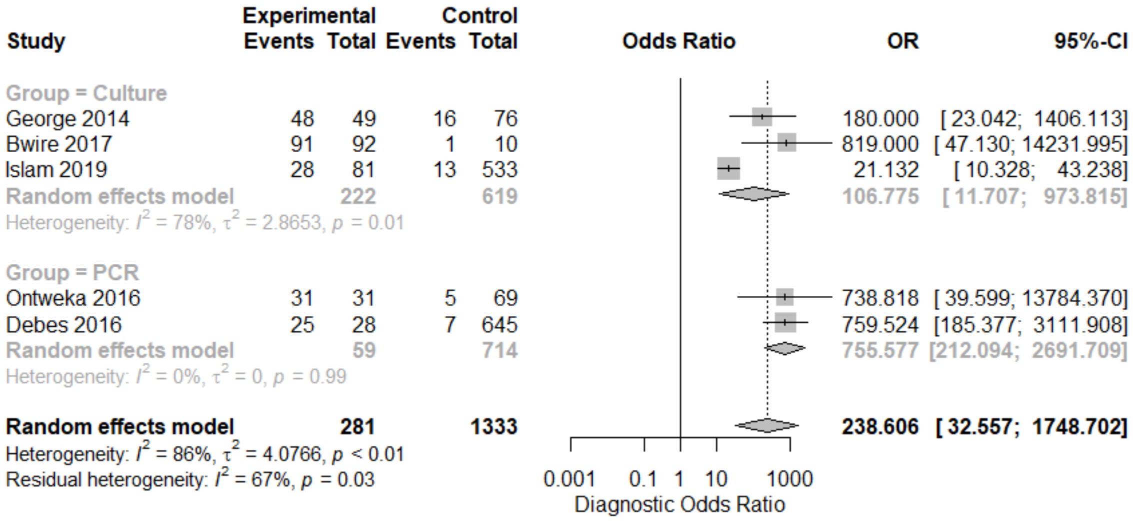

*Experimental: Events = True Positives; Total = True Positives + False Positives. Control: Events = False Negatives; Total = True Negatives + False Negatives

Figure 11. Forest plot results for Cholera Screen, DOR meta-analysis

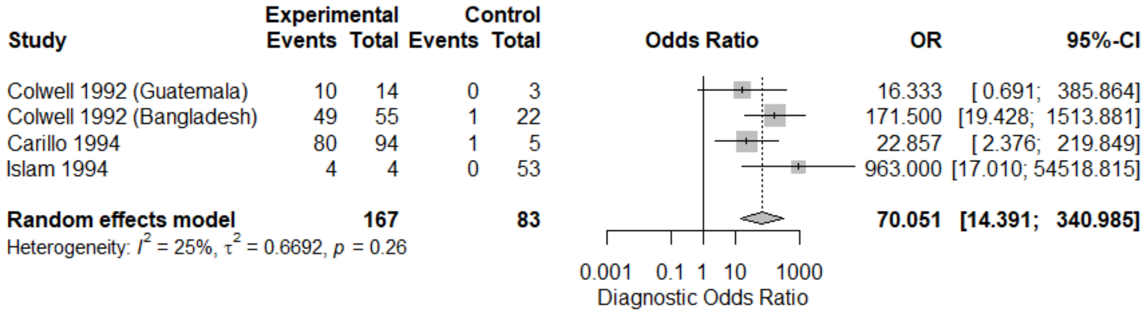

*Experimental: Events = True Positives; Total = True Positives + False Positives. Control: Events = False Negatives; Total = True Negatives + False Negatives

Figure 12. Forest plot results for IP Dipstick - Direct Samples, DOR meta-analysis

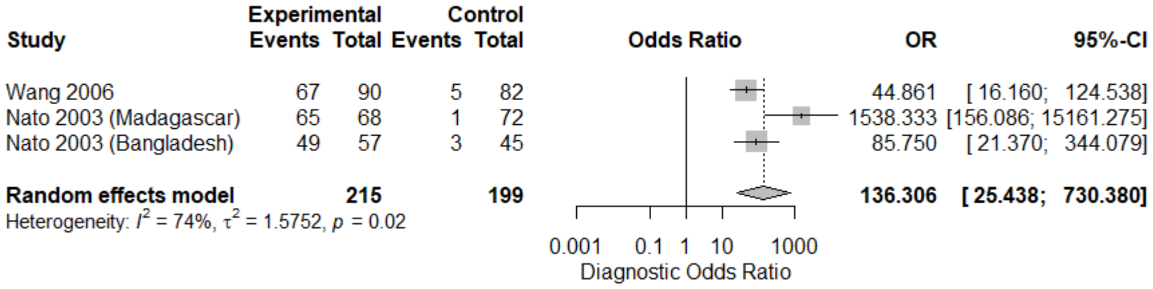

*Experimental: Events = True Positives; Total = True Positives + False Positives. Control: Events = False Negatives; Total = True Negatives + False Negatives
