# Supplementary material for: Cholera diagnosis in human stool and detection in water: A systematic review and meta-analysis
Source: PLoS One. 2022 Jul 6;17(7):e0270860. doi: 10.1371/journal.pone.0270860 (PMC9258865; doi:10.1371/journal.pone.0270860)

Appendix S4: Summary of Findings for individual studies

Immunologically-based tests: Crystal VC

Table 1. Sensitivity and specificity results for individual studies reporting on Crystal VC

**Test: Crystal VC (Institut Pasteur/Span Diagnostics)**				
**Intended Location: Field**				
**Target: lipopolysaccharide antigen of V. cholerae O1 and O139**				
**Reference test: Bacterial Culture**				
**Sample type: Stool**				
**Study ID**	**n**	**Enrichment details (if relevant)**	**Sensitivity**	**Specificity**
Direct Samples				
George 2014	125		65.6 (52.7-77.1)	91.8 (81.9-97.3)
Page 2012 (lab technicians)	256		93.0 (88.3-96.6)	85.2 (69.8-99.2)
Page 2012 (clinicians)	255		93.8 (89.2-97.2) [1,4]	78.4 (59.6-98.7) [1,4]
Ley 2012	622		93.1 (88.7-96.2) [4]	49.2 (44.3-54.1) [4]
Mukherjee 2010	212		91.70	72.90
Islam 2019	5865		72.2 (64.6-78.9)	77.1 (75.9-78.2)
Sayeed 2018	76		97.5 (87.5-99.9) [4]	98.4 (92.0-99.9) [4]
Matias 2017	511		98.6 (96.5-99.6)	71.1 (64.6-76.9)
Enriched samples				
George 2014	125	6h enrichment in APW	75 (62.6-85)	98.4 (91.2-100)
Bwire 2017	102	6h enrichment in APW	98.9 (94.09-99.97)	90 (55.5-99.75)
Islam 2019	614	Overnight enrichment in APW	68.3 (51.9-81.9)	90.8 (88.1-92.9)
**Sample type: Water**				
Enriched samples				
Rashid 2017 [2]	1648	18h enrichment in APW	65.6 (55.2-75)	99.6 (99.2-99.9)
Chakraborty 2013	550	24h incubation in APW	87 (74.9-94.3) [1]	100 (99-100) [1]

**Reference test: PCR**				
**Sample type: Stool**				
Direct Samples				
Ontweka 2016	100		94.4 (81.3-99.3)	79.7 (67.8-88.7)
Harris 2009	99		97 (88.7-99.5)	75 (56.2-87.9)[3]
Enriched Samples				
Ontweka 2016	100	4-6h enrichment in APW	86.1 (70.5-95.3)	100 (94.4-100)
Debes 2016	673	24h enrichment in APW	89.3 (71.8-97.7) [3]	98.9 (97.8-99.6) [3]
**Reference test: Culture or PCR**				
**Sample type: Stool**				
Direct Samples				
Page 2012 (lab technicians)	256		88.2 (82.6-92.4)	88.6 (78.7-94.9)
Page 2012 (clinicians)	255		91.9 (87-95.4)	82.6 (71.6-90.7)

[1]Confidence Intervals not provided; calculated from raw numbers
[2]Combined supply and stored water
[3]Sensitivity and specificity re-calculated using raw numbers due to errors in paper
[4] Estimated by Bayesian Latent Modelling

Figure 1. Plot of sensitivities and specificities for individual studies. Each dot represents a single study result, sub-grouped by colour according to 'reference test – sample type – enrichment status' as per the legend below. Error bars show 95% Confidence Intervals.

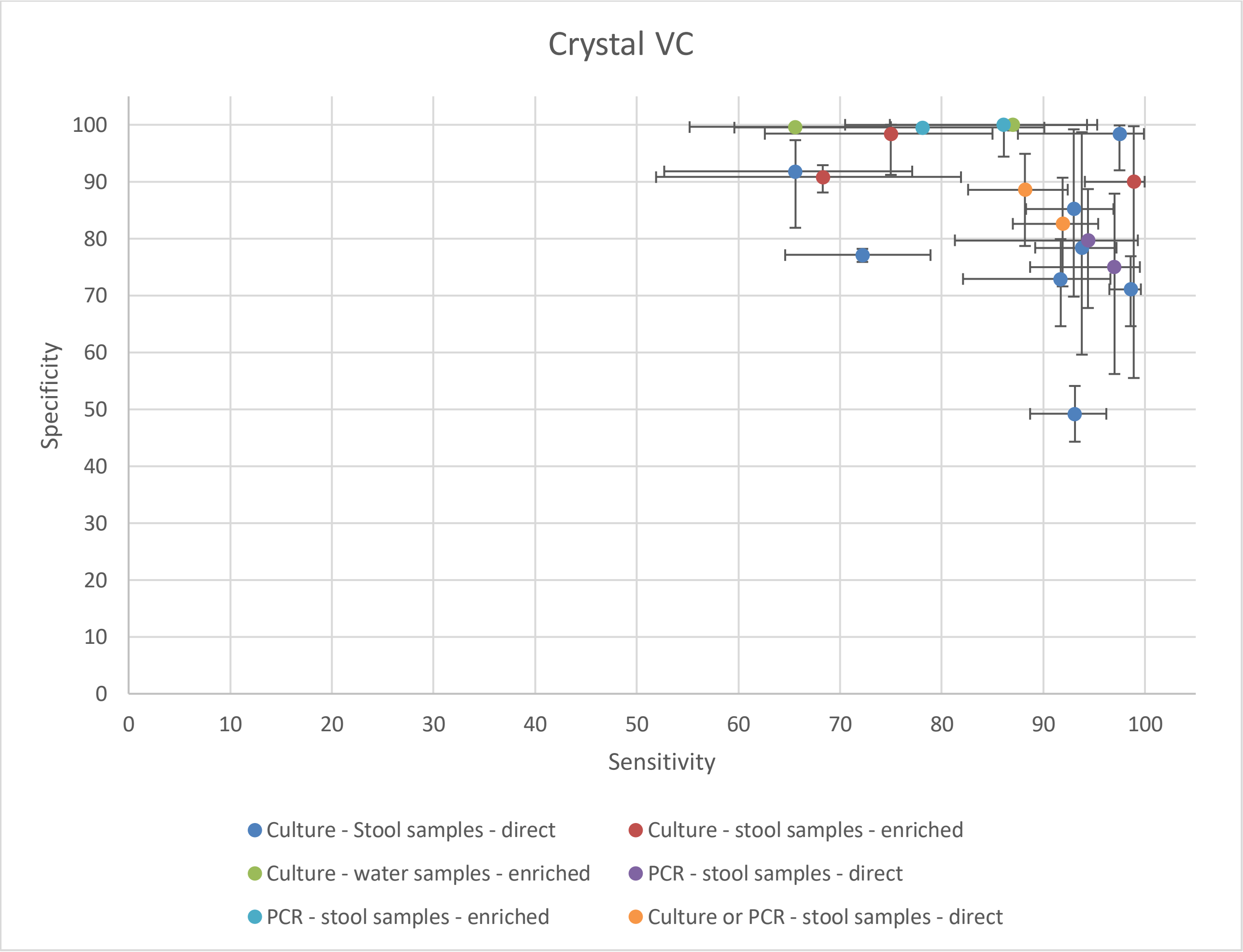

Immunologically-based tests: CholeraSMART

Table 2. Sensitivity and specificity results for individual studies reporting on Cholera SMART

**Test: Cholera SMART (New Horizons Diagnostics)**				
**Intended Location: Field**				
**Target: Antigen A of V. cholerae O1 lipopolysaccharide**				
**Reference test: Bacterial Culture**				
**Sample type: Stool**				
**Study ID**	**n**	**Enrichment details (if relevant)**	**Sensitivity**	**Specificity**
Direct Samples				
Bolaños 2004	28		100 (74.7-100) [1]	100 (71.7-100) [1]
Hasan 1994b (Bangladesh)	44		95.6 (76-99.8) [1]	100 (80.8-100) [1]
Kalluri 2006 (lab technicians)	254		83 (75-90)	88 (82-93)
Kalluri 2006 (field technicians)	212		58 (46-71)	95 (91-98)
**Reference test: Cholera Screen**				
**Sample type: Stool**				
Direct Samples				
Hasan 1994b (Mexico)	108		100 (90.2-100) [1]	100 (92.8-100) [1]

[1] Confidence Intervals not provided; calculated from raw numbers

Figure 2. Plot of sensitivities and specificities for individual studies. Each dot represents a single study result, sub-grouped by colour according to 'reference test – sample type – enrichment status' as per the legend below. Error bars show 95% Confidence Intervals

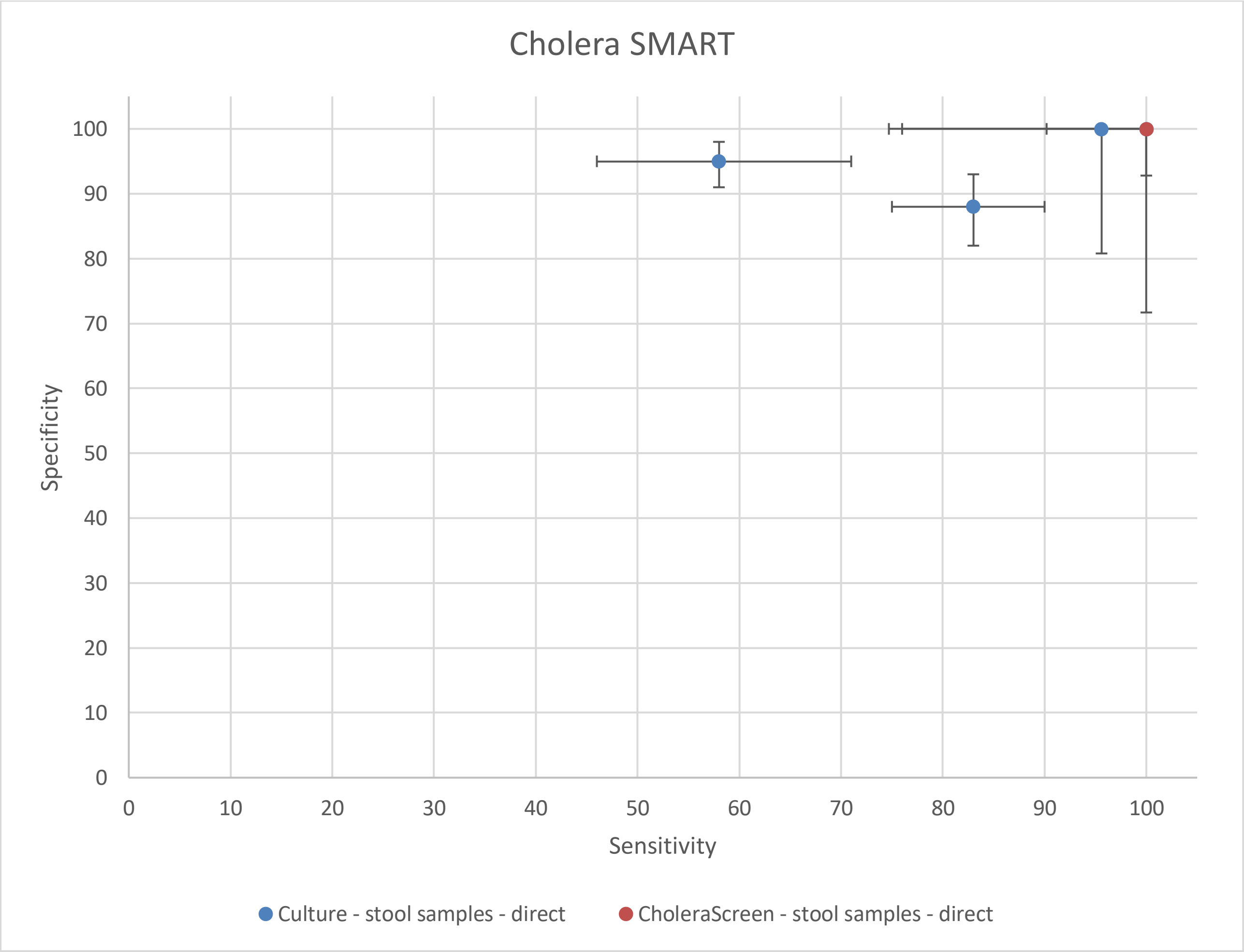

Immunologically-based tests: IP Dipstick

Table 3. Sensitivity and specificity results for individual studies reporting on IP Dipstick

**Test: IP Dipstick (Institut Pasteur)**				
**Intended Location: Field**				
**Target: V. cholerae O1 lipopolysaccharide**				
**Reference test: Bacterial Culture**				
**Sample type: Stool**				
**Study ID**	**n**	**Enrichment details (if relevant)**	**Sensitivity**	**Specificity**
Direct Samples				
Wang 2006	172		93 (87-99)	77 (69-85)
Kalluri 2006 (field technicians)	NR		93 (87-97)	67 (60-74)
Kalluri 2006 (lab technicians)	NR		94 (88-98)	76 (70-82)
Nato 2003 (Madagascar)	140		98.5 (90.7-99.9) [1]	95.9 (87.8-98.9) [1]
Nato 2003 (Bangladesh)	102		94.2 (83.1-98.5) [1]	84 (70.3-92.4) [1]
Enriched Samples				
Wang 2006	219	6h in APW	97 (93-100)	97 (95-100)
Bhuiyan 2003	133	4h in APW	97 (88.7-99.5) [1]	92.4 (82.5-97.2) [1]
**Target: V. cholerae O139 lipopolysaccharides**				
**Reference test: Bacterial Culture**				
**Sample type: Stool**				
Direct Samples				
Nato 2003 (Bangladesh)	158		100 (95-100) [1]	92.5 (82.7-97.2) [1]
Enriched Samples				
Bhuiyan 2003	134	4h in APW	92.6 (74.2-98.7) [1]	98.1 (92.8-99.7) [1]

[1] Confidence Intervals not provided; calculated from raw numbers

Figure 3. Plot of sensitivities and specificities for individual studies. Each dot represents a single study result, sub-grouped by colour according to 'serotype - reference test – sample type – enrichment status' as per the legend below. Error bars show 95% Confidence Intervals

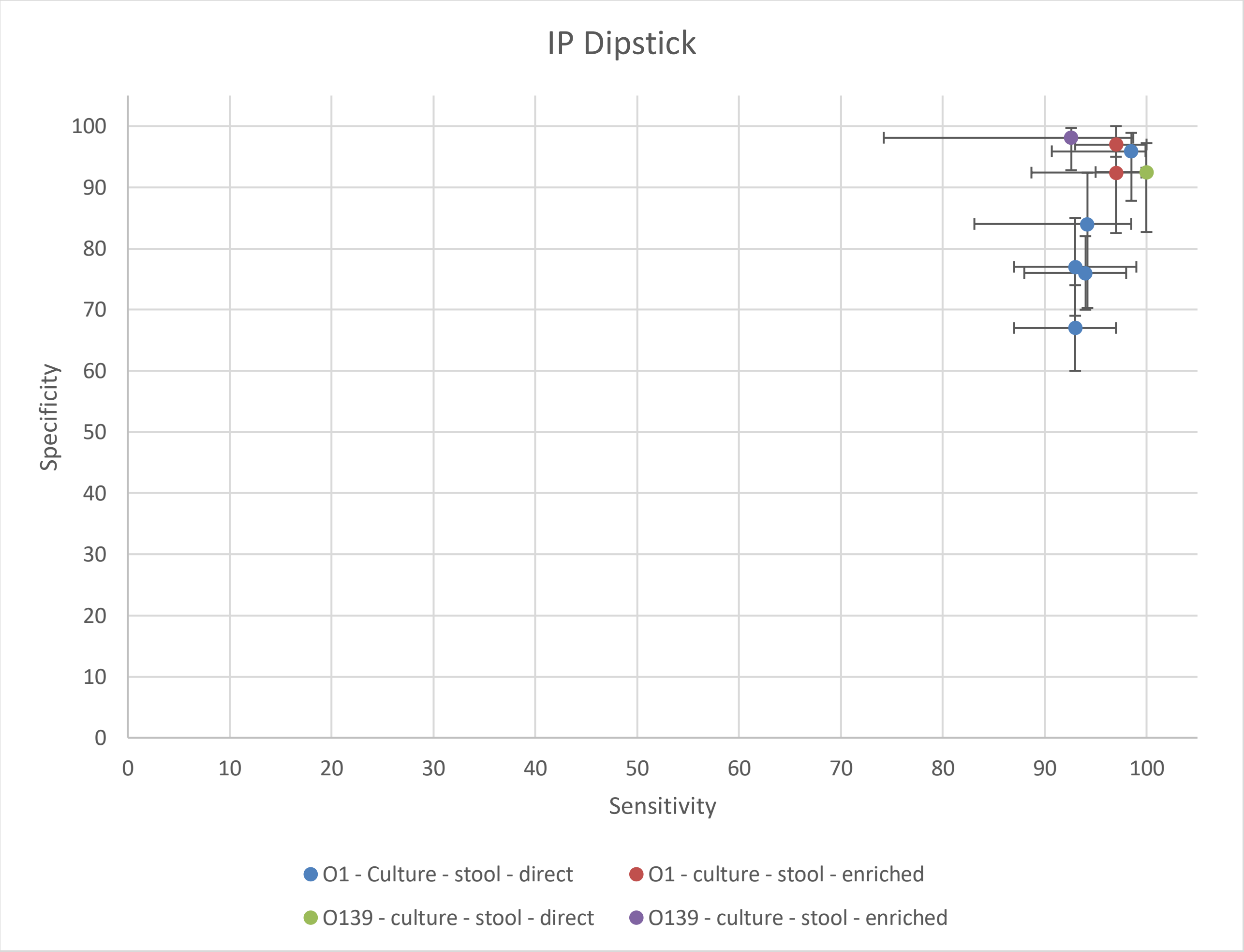

Immunologically-based tests: Cholera Screen

Table 4. Sensitivity and specificity results for individual studies reporting on Cholera Screen

Test: Cholera Screen				
**Intended Location: Field**				
**Target: 'A' factor of V. cholerae lipopolysaccharide O1**				
**Reference test: Bacterial Culture**				
**Sample type: Stool**				
**Study ID**	**n**	**Enrichment details (if relevant)**	**Sensitivity**	**Specificity**
Direct Samples				
Colwell 1992 (Guatemala)	17		100 (65.5-100) [1]	42.9 (11.8-79.8) [1]
Colwell 1992 (Bangladesh)	77		98 (88-99.9) [1]	77.8 (57.3-90.6) [1]
Carillo 1994	99		98.8 (92.4-99.9) [1]	22.2 (7.4-48.1) [1]
Islam 1994	57		100 (39.58-100) [1]	100 (91.58-100) [1]

[1] Confidence Intervals not provided; calculated from raw numbers

Figure 4. Plot of sensitivities and specificities for individual studies. Each dot represents a single study result, sub-grouped by colour according to 'reference test – sample type – enrichment status' as per the legend below. Error bars show 95% Confidence Intervals

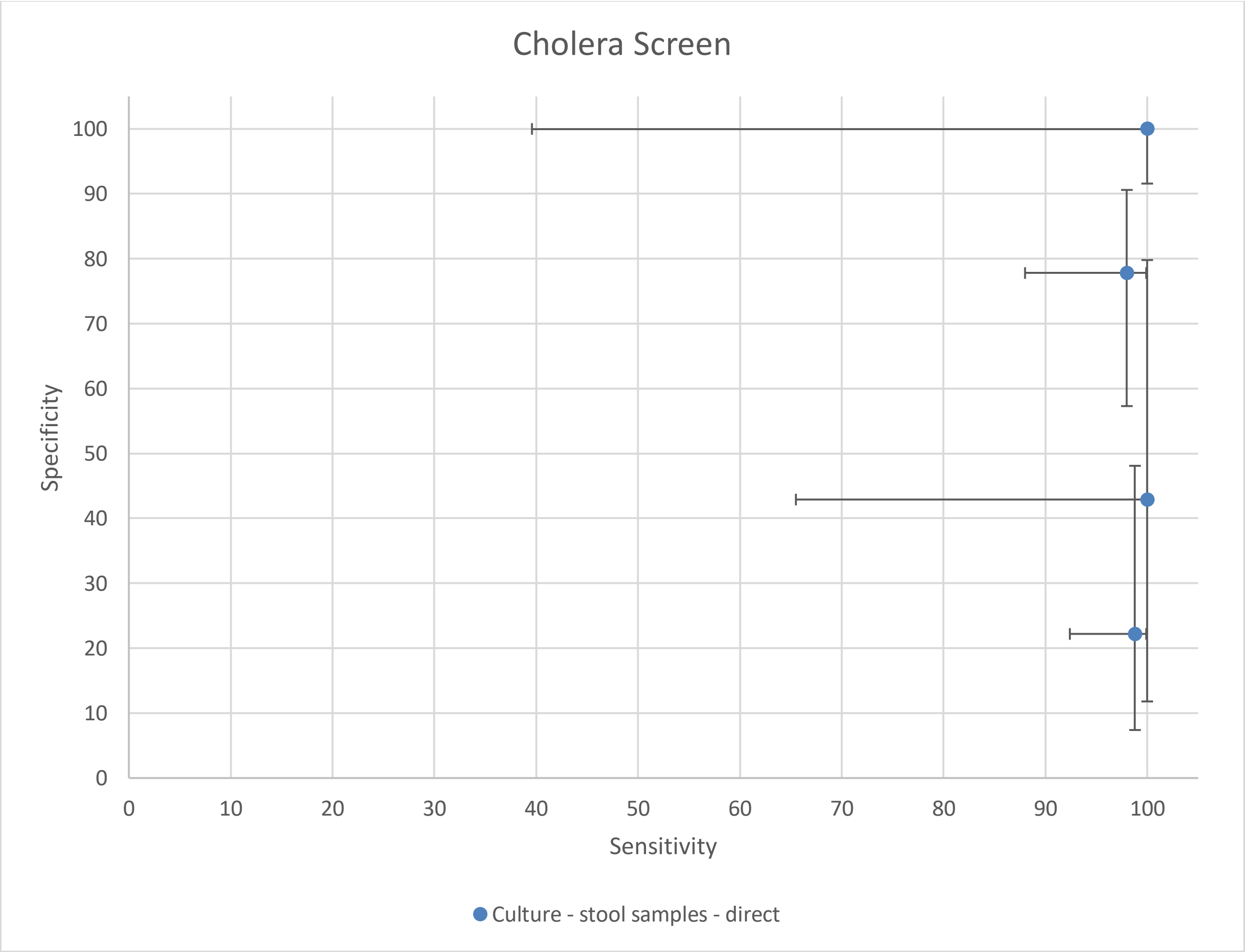

Immunologically-based tests: ELISA-based tests

Table 5. Sensitivity and specificity results for individual studies reporting on ELISA-based tests

**Test: ELISA-based tests - two-tip dipstick sandwich ELISA**				
**Intended Location: field**				
**Target: V. cholerae O1 and O139 (Stool); Ctx b and ompw of O1 and O139 (water)**				
**Reference test: Culture**				
**Sample type: Stool or water**				
**Study ID**	**n**	**Enrichment and sub-group details (if relevant)**	**Sensitivity**	**Specificity**
Enriched Samples				
Tuteja 2007 (Stool samples)	75	4h in APW	100 (91.4-100) [1]	100 (82.2-100) [1]
Tuteja 2007 (Water samples)	50	4h in APW	100 (31-100) [1]	100 (90.6-100) [1]
**Test: ELISA-based tests - Cholera diagnostic kit (Mab-based dot-blot ELISA)**				
**Intended Location: field**				
**Target: V cholerae O1 antigen A**				
**Reference test: Culture**				
**Sample type: Stool**				
Enriched Samples				
Supawat 1994 (Diarrhoeic patients)	211	4h in APW	100 (73.2-100) [1]	100 (97.6-100) [1]
Supawat 1994 (Household contacts)	415	4h in APW	95.2 (74.1-99.8) [1]	100 (98.8-100) [1]
**Test: ELISA-based tests - dot-blot ELISA**				
**Intended Location: laboratory**				
**Target: V. cholerae O1 antigen or V. cholerae O139 antigen**				
**Reference test: Culture**				
**Sample type: Stool**				
Direct Samples				
Chaicumpa 1995	147	O1 antigen	63 (53.1-71.3) [1]	97 (82-99.8) [1]

Enriched Samples				
Chaicumpa 1998	6497	O139 antigen	100 (89.6-100) [1]	99.95 (99.8-99.9) [1]
**Test: ELISA-based tests - bead ELISA**				
**Intended Location: laboratory**				
**Target: Cholera toxin**				
**Reference test: Culture**				
**Sample type: Stool**				
Direct Samples				
Ramamurthy 1996 (PAb-based)	95		82.1 (71.1-89.8) [1]	63.6 (40.8-82) [1]
Ramamurthy 1996 (MAb-based)	95		69.9 (57.9-79.8) [1]	81.8 (59-94) [1]
Ramamurthy 1993	123		82.9 (71.6-90.5) [1]	79.2 (65.5-88.7) [1]
Ramamurthy 1992	75		84.7 (72.5-92.5) [1]	81.3 (53.7-95) [1]

[1] Confidence Intervals not provided; calculated from raw numbers

Figure 5. Plot of sensitivities and specificities for individual studies. Each dot represents a single study result, sub-grouped by colour according to type of ELISA test, as per the legend below. Error bars show 95% Confidence Intervals

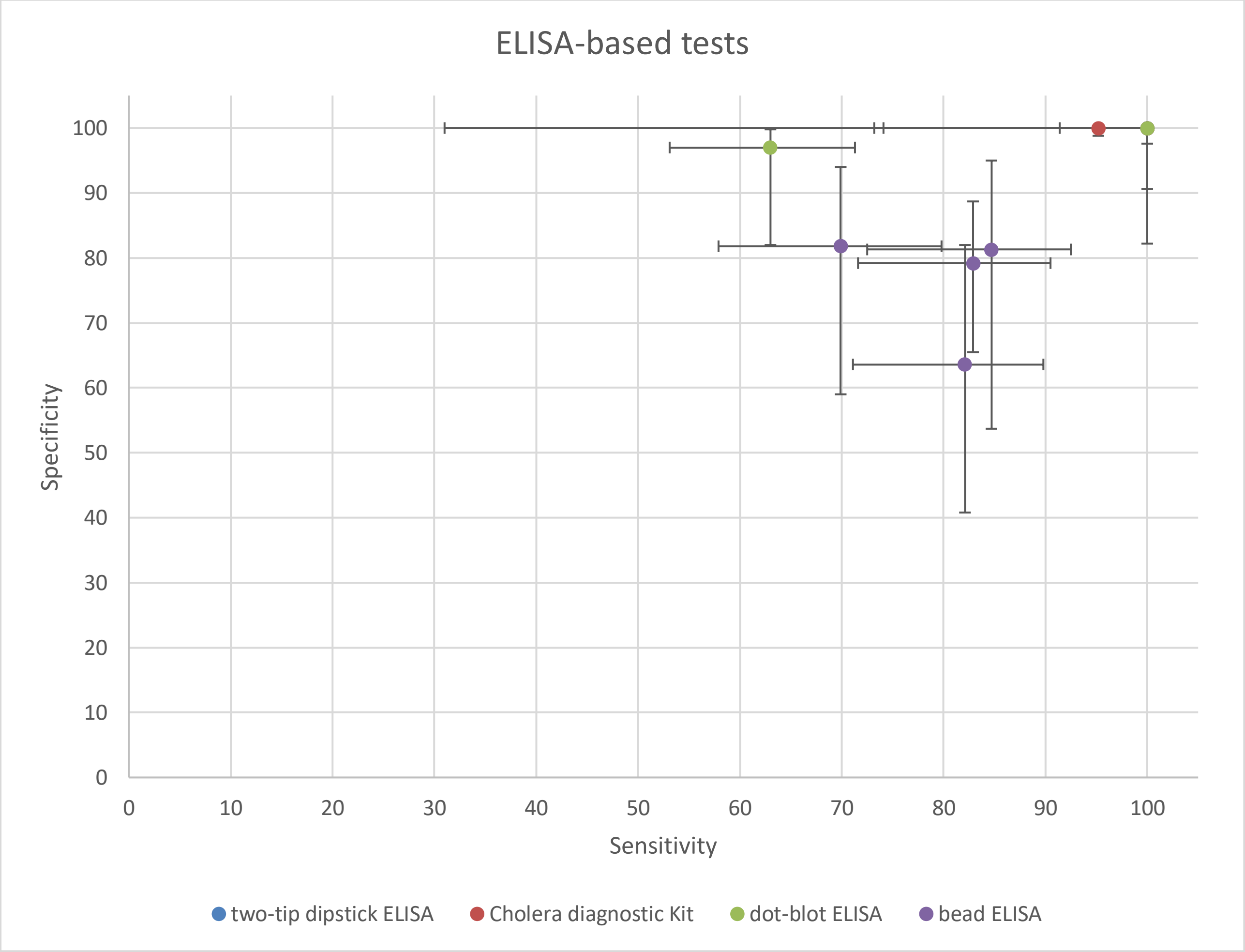

Other tests

Table 6. Sensitivity and specificity results for individual studies reporting on other tests not mentioned above

**Test: Other tests**						
**Intended Location: Field**						
**Reference test: Culture**						
**Sample type: Stool**						
**Study**	**Test name**	**Target**	**n**	**Enrichment and sub-group details (if relevant)**	**Sensitivity**	**Specificity**
Direct Samples						
Kalluri 2006	Medicos Cholera Dip Stick	Unknown	304	Field technicians	84 (77-91)	79 (73-85)
Kalluri 2006	Medicos Cholera Dip Stick	Unknown	303	Lab technicians	88 (81-94)	80 (73-95)
Bolaños 2004	PDK (Pathogen Detection Kit)	Antigen A of V. cholerae O1 lipopolysaccharide	27	High probability specimens	100 (71.7-100) [1]	86 (56.2-97.5) [1]
Qadri 1995	Bengal SMART	A' factor of V. cholerae O139	189		97 (89.5-99.1) [1]	100 (95.5-100) [1]
Hasan 1995	BengalScreen	lipopolysaccharide antigen of V. cholerae O139	35		95 (71.9-99.7) [1]	100 (75.9-100) [1]
Hasan 1995	Bengal DFA	lipopolysaccharide antigen of V. cholerae O139	35		100 (79.1-100) [1]	100 (75.9-100) [1]
Qadri 1994	co-agglutination test (COAT)	lipopolysaccharide antigens of V. cholerae O139	230		92 (84.1-96.5) [1]	100 (96.7-100) [1]
Carillo 1994	latex agglutination test	A' factor of V. cholerae lipopolysaccharide O1	100		100 (94.4-100) [1]	33 (14.4-58.8) [1]
Islam 2019	Cholkit	lipopolysaccharide antigen of V. cholerae O1	1355		79.4 (62.1-91.3)	87.4 (85.5-89.1)
Sayeed 2018	Cholkit	lipopolysaccharide antigen of V. cholerae O1	76		97.7 (88.4-99.9) [2]	96.5 (88.6-99.6) [2]
Matias 2017	Artron RDT	Vibrio cholerae O1 and O139	129		98.6 (92.7-100)	69.1 (55.2-80.9)
Matias 2017	SD Bioline	V. choleraeO1 and O139 antigens	451		81.1 (75.6-85.8)	92.8 (88.4-95.9)

Enriched Samples						
Islam 2019	Cholkit	lipopolysaccharide antigen of V. cholerae O1	424		66.7 (47.2-82.7)	94.4 (91.7-96.5)
**Reference test: Culture plus PCR**						
**Sample type: Stool**						
Direct Samples						
Mwaba 2018	SD Bioline	V. choleraeO1 and O139 antigens	*170*		90.9 (81.3-96.6)	95.2 (89.1-98.4)
Enriched Samples						
Mwaba 2018	SD Bioline	V. choleraeO1 and O139 antigens	*170*		95.5 (87.3-99.1)	100 (96.5-100)
**Reference test: Combination**						
**Sample type: Water**						
Direct Samples						
Hao 2017	Vch-UPT-LF	V. cholerae O1 or O139	96	O1	100 (71.7-100) [1]	100 (94.5-100) [1]
Hao 2017	Vch-UPT-LF	V. cholerae O1 or O139	96	O139	100 (19.8-100) [1]	99 (93.4-99.9) [1]
**Test: Other tests**						
**Intended Location: Laboratory**						
**Reference test: Culture**						
**Sample type: Stool**						
Direct Samples						
Hasan 1994a	Cholera DFA	A' factor of V. cholerae lipopolysaccharide O1	44		100 (82.2-100) [1]	100 (80.8-100) [1]
**Reference test: Combination**						
**Sample type: Stool**						
Direct Samples						
Jin 2013	CT-RTCA (cholera toxin real-time cell analysis)	Cholera toxin	100		90 (72.3-97.4) [1]	97 (89.1-99.5) [1]
Eddabra 2011	ChromID Vibrio	V. cholerae bacterial strains	30		79 (48.8-94.3) [1]	100 (75.9-100) [1]

Eddabra 2011	TCBS	V. cholerae bacterial strains	30		79 (48.8-94.3) [1]	50 (25.5-74.5) [1]
Enriched Samples						
Eddabra 2011	ChromID Vibrio	V. cholerae bacterial strains	*30*	Yes - 5-8h in APW	100 (73.2-100) [1]	100 (75.9-100) [1]
Eddabra 2011	TCBS	V. cholerae bacterial strains	30	Yes - 5-8h in APW	100 (73.2-100) [1]	50 (25.5-74.5) [1]

[1] Confidence Intervals not provided; calculated from raw numbers

[2] Result estimated using Bayesian Latent Modelling

Figure 6. Plot of sensitivities and specificities for individual studies. Each dot represents a single study result, sub-grouped by colour according to type test, as per the legend below. Error bars show 95% Confidence Intervals

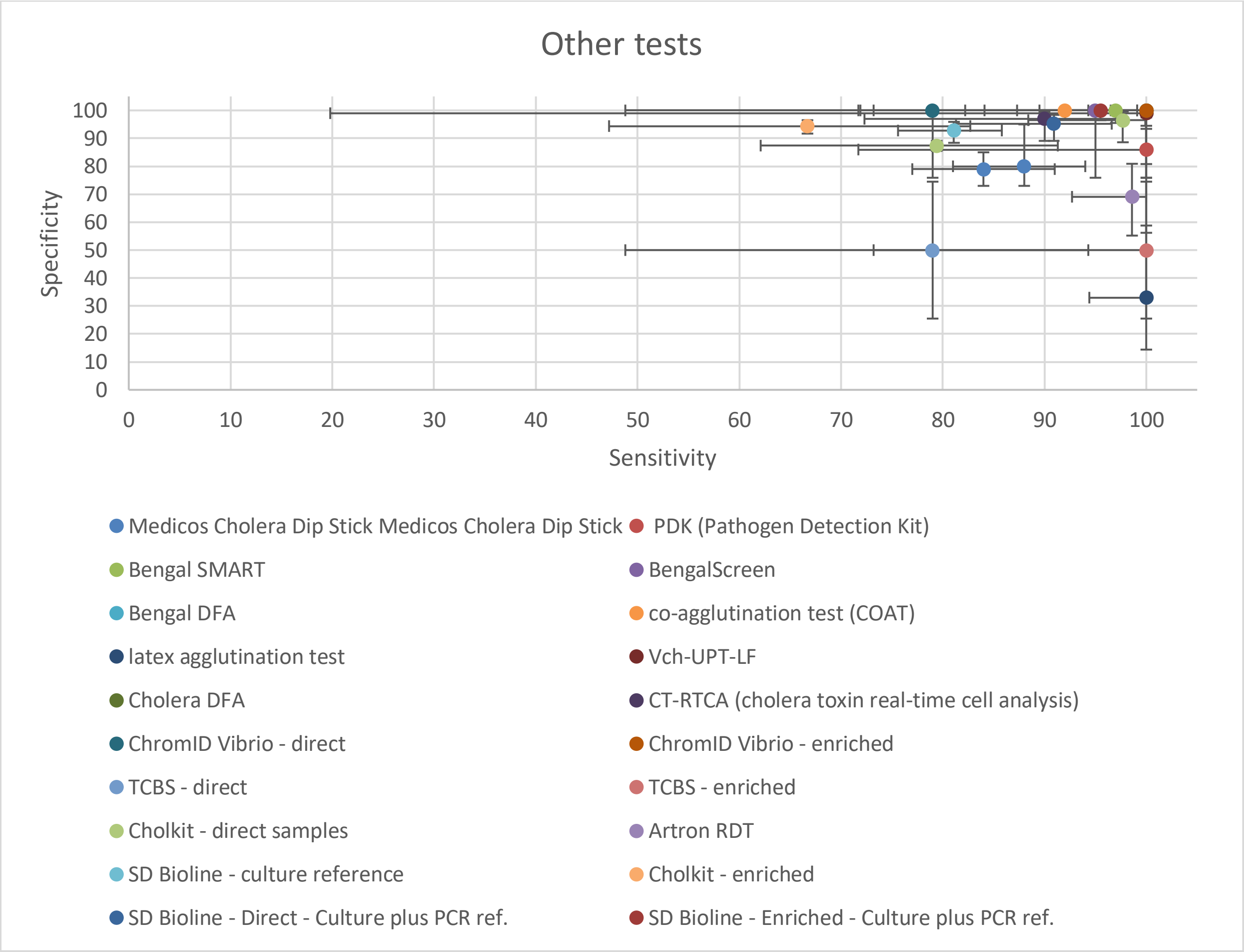

PCR-based tests

Table 7. Sensitivity and specificity results for individual studies reporting on PCR-based tests

**Test: PCR-based tests**						
**Intended Location: laboratory**						
**Reference test: Culture**						
**Sample type: Stool**						
**Study**	**Test name**	**Target**	**n**	**Enrichment and sub-group details (if relevant)**	**Sensitivity**	**Specificity**
Direct Samples						
Albert 1997	PCR assay (with new primers O139-1 and O139-2)	V. cholerae O139	180	Undiluted sample	94 (84.7-98.1) [1]	100 (95.9-100) [1]
Sayeed 2018	Multiplex PCR	V. cholerae O1 and O139 specific rfb genes and cholera toxin gene ctxA	76		73.6 (58.5-85.7) [2]	97.2 (93.2-99.2) [2]
Enriched Samples						
Hoshino 1998	Multiplex PCR (with: O139-rfb primers - O139F-2, O139R-2; O1-rfb primers - O1F-2, O1R-2; cholera toxin primers - VCT1, VCT2)	V. cholerae O1 and O139 rbf-specific genes and the ctxA gene.	121	18h in APW	100 (88.6-100) [1]	95 (87.5-98.4) [1]
Ramamurthy 1993	PCR (with primers specific to V. cholera O1)	Cholera Toxin gene of V. cholerae O1.	123	18h in APW	100 (93.5-100) [1]	55 (40.6-68.2) [1]
**Reference test: Culture**						
**Sample type: Water**						
Enriched Samples						
Momtaz 2013	PCR (with primers specific to espM gene)	epsM gene of V. cholerae	448	6-8h in APW	100 (31-100) [1]	100 (98.9-100) [1]
**Reference test: Conventional assay**						
**Sample type: Stool**						
Direct Samples						

Liu 2013	Taqman array Card	toxR gene	80		100 (59.8-100) [1]	100 (93.7-100) [1]
**Reference test: PCR Luminex**						
**Sample type: Stool**						
Direct Samples						
Liu 2013	Taqman array Card	toxR gene	109		100 (62.9-100) [1]	100 (95.4-100) [1]

[1] Confidence Intervals not provided; calculated from raw numbers

[2] Result estimated using Bayesian Latent Modelling

Figure 7. Plot of sensitivities and specificities for individual studies. Each dot represents a single study result, sub-grouped by colour according to type PCR test and reference or enrichment status, as per the legend below. Error bars show 95% Confidence Intervals.

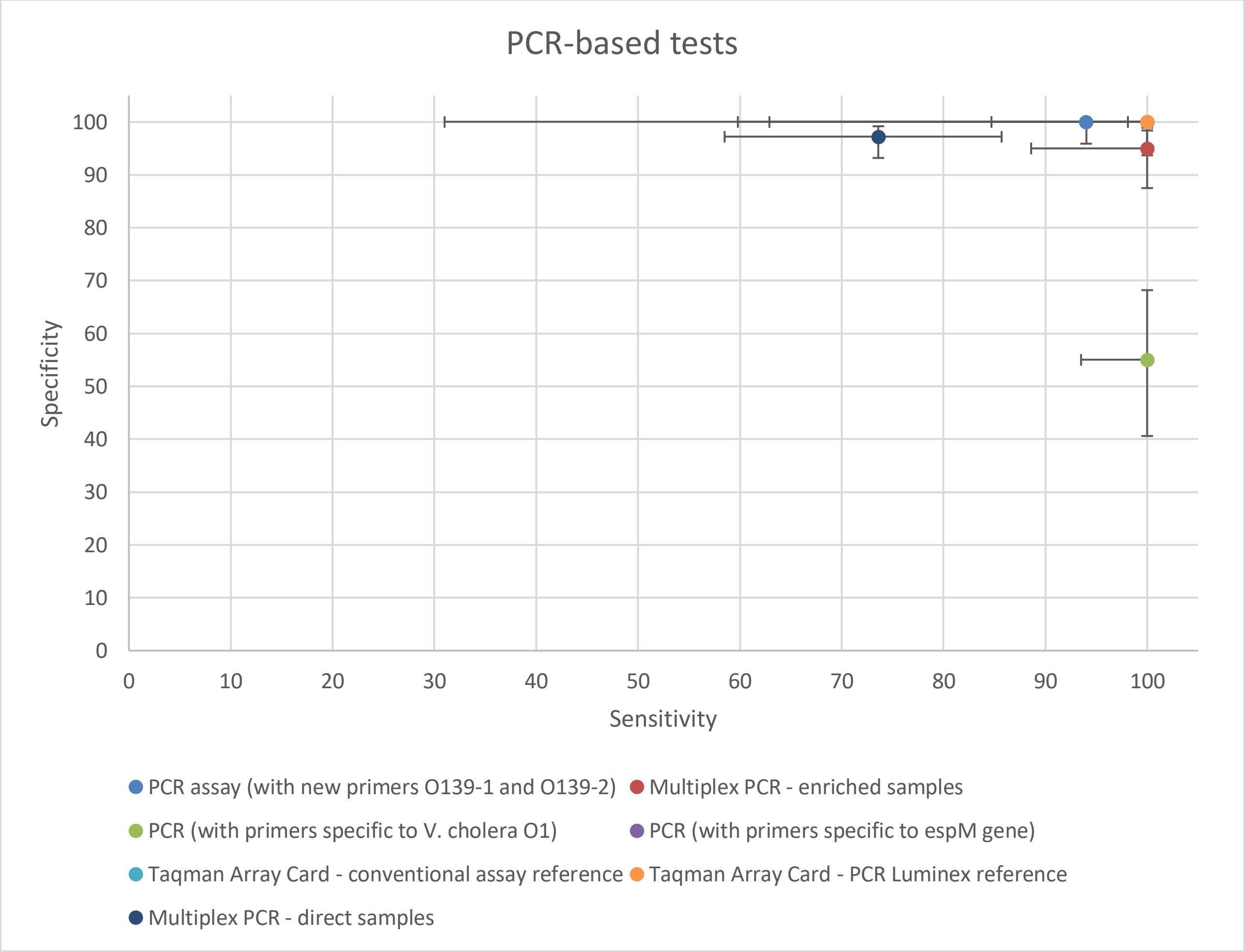

Supplement: S4 Appendix — (PDF) [file pone.0270860.s004.pdf]
