# Supplementary material for: Cholera diagnosis in human stool and detection in water: A systematic review and meta-analysis
Source: PLoS One. 2022 Jul 6;17(7):e0270860. doi: 10.1371/journal.pone.0270860 (PMC9258865; doi:10.1371/journal.pone.0270860)
Supplement: S6 Appendix — (PDF) [file pone.0270860.s006.pdf]

Appendix S6: Risk of bias and applicability – results by study

Study ID	Sample selection	Index test	Reference test	Sample flow
	Could the sample selection have introduced bias?	Could the interpretation of the index test have introduced bias?	Could the reference standard, its conduct or its interpretation have introduced bias?	Could the sample flow have introduced bias?
Albert 1997 [19]	Unclear	Low	Low[1]	Low
Bhuiyan 2003 [20]	Low	High	Low[1]	Low
Bolaños 2004 [21] (Cholera SMART index test)	Unclear	High	Low[1]	Low
Bolaños 2004 [21] (Pathogen Detection Kit index test)	Unclear	High	Low[1]	Low
Bwire 2017 [22]	Low	High	Low[1]	Low
Carillo 1994 [23] (Cholera Screen index test)	Unclear	High	Low[1]	Low
Carillo 1994 [23] (latex agglutination index test)	Unclear	High	Low[1]	Low
Chaicumpa 1995 [24]	Unclear	Low	Low[1]	Low
Chaicumpa 1998 [25]	Unclear	Low	Low[1]	Low
Chakraborty 2013 [26]	Unclear	High	Low[1]	Low
Colwell 1992 [27]	Unclear	Low	Low[1]	Low
Debes 2016 [28]	Low	Low	Low	High
Eddabra 2011 [29] (ChromID Vibrio index test)	Unclear	Low	Unclear	Low
Eddabra 2011 [29] (TCBS index test)	Unclear	Low	Unclear	Low
George 2014 [30]	Low	Low	Low	Low
Hao 2017 [31]	Unclear	High	Unclear	Low
Harris 2009 [32]	Low	Low	Low	Low
Hasan 1994a [33]	Low	Low	Low[1]	Low
Hasan 1994b [34] (Culture reference)	Unclear	Low	Low[1]	Low
Hasan 1994b [34] (Cholera Screen reference)	Unclear	Low	Low	Low

Hasan 1995 [35] (BengalScreen index test)	Low	Unclear	Low[1]	Low
Hasan 1995 [35] (BengalDFA index test)	Low	Unclear	Low[1]	Low
Hoshino 1998 [36]	Unclear	Low	Low[1]	Low
Islam 1994 [37]	Unclear	High	Low[1]	Low
Islam 2019 [38] (Crystal VC)	Low	Low	Low[1]	Low
Islam 2019 [38] (Cholkit)	Low	Low	Low[1]	Low
Jin 2013 [39]	Unclear	Low	Unclear	Low
Kalluri 2006 [40] (Cholera SMART index test)	Low	Low	Low[1]	Low
Kalluri 2006 [40] (IP dipstick index test)	Low	Low	Low[1]	Low
Kalluri 2006 [40] (Medicos Cholera dipstick index test)	Low	Low	Low[1]	Low
Ley 2012 [41]	Low	Low	Low[1]	Low
Liu 2013 [42] (conventional assay reference)	Unclear	Low	Low	High
Liu 2013 [42] (PCR Luminex reference)	Unclear	Low	Low	Low
Matias 2017 [43] (Artron RDT)	Low	High	Low[1]	Low
Matias 2017 [43] (Crystal VC)	Low	High	Low[1]	High
Matias 2017 [43] (SD Bioline)	Low	High	Low[1]	High
Momtaz 2013 [44]	Unclear	Low	Low[1]	Low
Mukherjee 2010 [45]	Low	Low	Low[1]	Low
Mwaba 2018 [46]	Low	Low	Low	Low
Nato 2003 [47]	Unclear	High	Low[1]	Low
Ontweka 2016 [10]	Low	Low	Low	Low
Page 2012 [48] (culture reference)	Low	Low	Low[1]	Low
Page 2012 [48] (culture or PCR reference)	Low	Low	Low	Low
Qadri 1994 [49]	Unclear	High	Low[1]	Low
Qadri 1995 [50]	Unclear	High	Low[1]	Low
Ramamurthy 1992 [51]	Low	Low	Low[1]	Low
Ramamurthy 1993 [52] (PCR index test)	Low	Low	Low[1]	Low
Ramamurthy 1993 [52] (Bead-ELISA index test)	Low	Low	Low	Low
Ramamurthy 1996 [53]	Unclear	Low	Low[1]	Low

Rashid 2017 [1]	Low	Unclear	Low[1]	Low
Sayeed 2018 [54] (Cholkit)	Unclear	High	Low[1]	Low
Sayeed 2018 [54] (Crystal VC)	Unclear	High	Low[1]	Low
Sayeed 2018 [54] (Multiplex PCR)	Unclear	Low	Low[1]	Low
Supawat 1994 [55]	Unclear	Low	Low[1]	Low
Tuteja 2007 [56]	Low	Unclear	Low	Low
Wang 2006 [57]	Low	High	Low[1]	Low
